# Connections between the human gut microbiome and gestational diabetes mellitus

**DOI:** 10.1093/gigascience/gix058

**Published:** 2017-07-31

**Authors:** Ya-Shu Kuang, Jin-Hua Lu, Sheng-Hui Li, Jun-Hua Li, Ming-Yang Yuan, Jian-Rong He, Nian-Nian Chen, Wan-Qing Xiao, Song-Ying Shen, Lan Qiu, Ying-Fang Wu, Cui-Yue Hu, Yan-Yan Wu, Wei-Dong Li, Qiao-Zhu Chen, Hong-Wen Deng, Christopher J. Papasian, Hui-Min Xia, Xiu Qiu

**Affiliations:** 1 Division of Birth Cohort Study, Guangzhou Women and Children's Medical Center, Guangzhou Medical University, 9 Jinsui Road, Guangzhou 510623, China; 2 Department of Women and Children's Health Care, Guangzhou Women and Children's Medical Center, Guangzhou Medical University, 9 Jinsui Road, Guangzhou 510623, China; 3 BGI-Shenzhen, China National GeneBank-Shenzhen, Dapeng District, Shenzhen 518083, China; 4 Shenzhen Key Laboratory of Human commensal microorganisms and Health Research, BGI-Shenzhen, Dapeng District, Shenzhen 518083, China; 5 Department of Obstetrics and Gynecology, Guangzhou Women and Children's Medical Center, Guangzhou Medical University, 9 Jinsui Road, Guangzhou 510623, China; 6 Center of Bioinformatics and Genomics, Department of Biostatistics and Bioinformatics, Tulane School of Public Health and Tropic Medicine, New Orleans, LA, 1010 Wayne Avenue, Suite 220, USA; 7 Department of Basic Medical Science, School of Medicine, University of Missouri – Kansas City, 2411 Holmes St., Kansas City, MO 64108, USA; 8 Department of Neonatal Surgery, Guangzhou Women and Children's Medical Center, Guangzhou Medical University, 9 Jinsui Road, Guangzhou 510623, China

**Keywords:** gut microbiome, gestational diabetes mellitus, metagenome-wide association

## Abstract

The human gut microbiome can modulate metabolic health and affect insulin resistance, and it may play an important role in the etiology of gestational diabetes mellitus (GDM). Here, we compared the gut microbial composition of 43 GDM patients and 81 healthy pregnant women via whole-metagenome shotgun sequencing of their fecal samples, collected at 21–29 weeks, to explore associations between GDM and the composition of microbial taxonomic units and functional genes. A metagenome-wide association study identified 154 837 genes, which clustered into 129 metagenome linkage groups (MLGs) for species description, with significant relative abundance differences between the 2 cohorts. *Parabacteroides distasonis, Klebsiella variicola*, etc., were enriched in GDM patients, whereas *Methanobrevibacter smithii, Alistipes* spp., *Bifidobacterium* spp., and *Eubacterium* spp. were enriched in controls. The ratios of the gross abundances of GDM-enriched MLGs to control-enriched MLGs were positively correlated with blood glucose levels. A random forest model shows that fecal MLGs have excellent discriminatory power to predict GDM status. Our study discovered novel relationships between the gut microbiome and GDM status and suggests that changes in microbial composition may potentially be used to identify individuals at risk for GDM.

## Background

The increasing prevalence of gestational diabetes mellitus (GDM), and its subsequent health outcomes, is a significant public health concern and a major challenge for obstetric practice [1]. GDM represents a heterogeneous group of metabolic disorders [2] that affects 3–14% of pregnancies, and 20–50% of these affected women are expected to develop type 2 diabetes (T2D) within 5 years [3, 4]. Emerging evidence has revealed a link between the gut microbiome and human metabolic health including T2D [5, 6], leading us to hypothesize that the gut microbiome may impact gestational metabolism and development of GDM.

Microbial dysbiosis in the human gut may be an important environmental risk factor for abnormal host metabolism, as recently exemplified in studies of obesity and T2D (reviewed by Karlsson et al.) [7]. A study using an experimental animal model revealed that reduced numbers of *Bifidobacteria* led to enhanced endogenous lipopolysaccharide production, endotoxemia, and associated obesity and insulin resistance [8]. In humans, excessive weight gain and obesity in pregnancy resulted in deteriorated glucose tolerance and increased risk of GDM [9, 10]. *Prevotella copri* and *Bacteroides vulgatus* have been identified as the main species driving the association between biosynthesis of branched-chain amino acids, insulin resistance, and glucose intolerance [11]. *Bacteroides* spp. and *Staphylococcus aureus* are significantly more abundant in overweight women than in normal-weight women [12].

While the majority of previous studies have focused on associations between intestinal microbiota and obese states or T2D [6, 13–15], some recent studies have sought to characterize microbiota changes during pregnancy, with the goal of providing novel insights into the relationship between microbiota changes during pregnancy and potential metabolic consequences [16]. Studies based on sequencing of 16S ribosomal RNA have revealed novel relationships between gut microbiome composition and the metabolic hormonal environment in overweight and obese pregnant women in early gestation [17]. Koren et al. found that maternal gut microbiota changed from the first to third trimesters, with a decline in butyrate-producing bacteria and increased *Bifidobacteria, Proteobacteria*, and lactic acid–producing bacteria [16]. Further, transplants of fecal material obtained during different trimesters were sufficient to confer different phenotypes in mouse models, with third-trimester fecal transplants leading to increased adiposity and inflammation [16]. These studies suggest that pregnancy is associated with major shifts in the gut microbiome that may play an important role in observed increases in gestational inflammation, thereby potentially contributing to the development of GDM. However, studies focusing on changes in the gut microbiome during pregnancy and the development of GDM have not been reported so far.

Metagenomic shotgun sequencing, in which the full complement of genes present in the microbiome are sequenced, can furnish information about the relative abundance of genes in functional pathways and at all taxonomical levels [18]. In this study, we used whole-metagenome shotgun sequencing analyses of the gut microbiome during pregnancy to explore associations between GDM and the composition and abundance of microbial taxonomic units and functional genes. The objective was to obtain a comprehensive understanding of the connections between the gut microbiome and the development of GDM.

## Data description

Whole-metagenome shotgun sequencing was used to test gut microbial composition in fecal samples from 43 GDM patients and 81 healthy pregnant women based on the Illumina HiSeq2000 platform in BGI-Shenzhen, China. We constructed a paired-end library with an insert size of 350 base pairs (bp) for every sample, sequenced with 100-bp read length from each end. Sequencing reads for fecal samples were independently processed for quality control and host sequence removal based on an in-house pipeline (see the Methods section), and a total of 795 Gbp of high-quality metagenomic data (average per sample, 6.4 Gbp) were generated for further analysis. We performed *de novo* assembly and gene calling for data from each sample and constructed a non-redundant gene catalogue of all pregnant women fecal samples containing 4 344 984 genes. This gene catalogue provided a suitable reference for metagenomic gene quantification, microbial diversity analysis, and metagenome-wide association study for the pregnant women fecal samples.

## Results

### Comparison of the gut microbiota between GDM patients and healthy pregnant women

**Figure 1:**
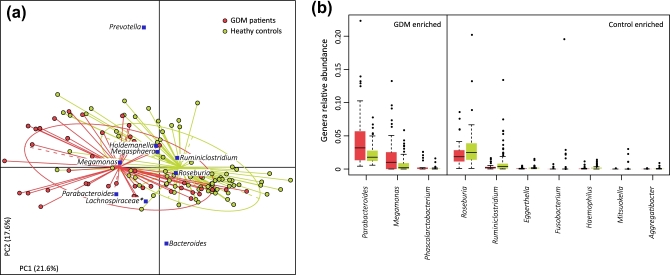
Difference in microbial composition between GDM and healthy pregnant women. (**a**) Distance-based redundancy analysis based on Bray–Curtis distances between microbial genera, revealing a GDM dysbiosis that overlaps only in part with taxonomic composition in GDM patients and healthy controls. The first 2 principal components (PCs) and the ratio of variance contributed by them is shown. Lines connect samples in the same group, and colored circles cover the samples near the center of gravity for each group. Genera (blue square), as the main contributors, are plotted by their loading in the PCs. (**b**) Boxplot shows genera that differ significantly between GDM patients and healthy controls. Genera with *q* < 0.05 (Mann–Whitney U test corrected by the Benjamini–Hochberg method) are shown. Red and green boxes represent GDM patients and healthy controls, respectively. Only the genera with average relative abundances greater than 0.05% in all the samples are shown for clarity. The boxes represent the interquartile range (IQR) between the first and third quartiles, and the line inside represents the median. The whiskers denote the lowest and highest values within 1.5 times IQR from the first and third quartiles, respectively. The circles represent outliers beyond the whiskers.

First, we explored potential differences in the gut microbiome between 43 GDM patients and 81 healthy pregnant women. We obtained 795.3 Gb of high-quality data (6.4 ± 1.3 Gb per sample) via metagenomic shotgun sequencing of their fecal samples to perform this analysis. When we quantified the microbial (alpha) diversity within each subject, the GDM patients showed significantly lower gene count and Shannon index compared with the healthy pregnant women (*P* < 0.05 for both indexes, Mann–Whitney U test).We then aligned the sequencing reads (43.8%) against available microbial genomes from the National Center for Biotechnology Information and generated taxonomic composition for all samples at the taxonomic levels of phylum, class, order, family, genus, and species. Multivariate analysis based on Bray–Curtis distances between microbial genera revealed significant differences between GDM patients and healthy controls (Fig. 1a). We then performed the Mann–Whitney U test to identify phylogenetic differences between GDM patients and healthy controls. Abundance at the phylum and class levels was similar between GDM patients and healthy controls; however, the order *Clostridiales* and the family *Coriobacteriaceae* were enriched in healthy controls. At the genus level, GDM patients had a significantly higher abundance of *Parabacteroides, Megamonas*, and *Phascolarctobacterium*, while healthy controls were significantly enriched for *Ruminiclostridium, Roseburia, Eggerthella, Fusobacterium, Haemophilus, Mitsukella*, and *Aggregatibacter* (Fig. 1b). We also found a number of bacterial species that differed significantly between GDM patients and healthy controls, consistent with the genus-level observations (Table S2). These findings suggest dysbiosis of the gut microbiota among GDM patients.

### Identification of GDM-associated markers from the gut microbiome

**Figure 2:**
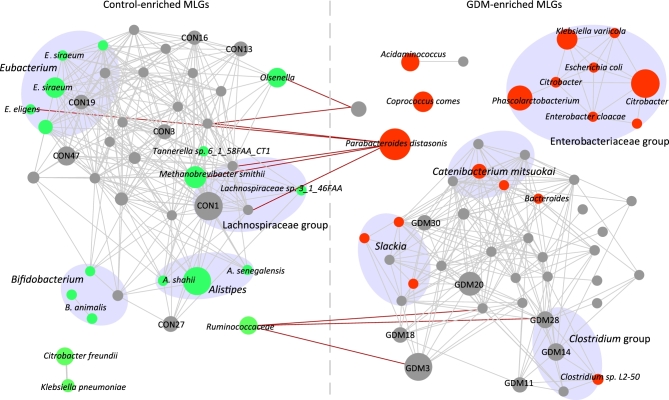
Interconnection of GDM-associated MLGs. A co-occurrence network deduced from GDM-enriched and control-enriched MLGs is shown. Nodes depict MLGs with their taxonomic assignment or ID shown. The size of each node indicates the number of genes within the MLG. Connecting lines represent Spearman correlation coefficient ρ > 0.40 (gray line) or < −0.40 (red line). Classified MLGs are colored (red: GDM-enriched; green: control-enriched) and grouped according to their taxonomic information. Only MLGs with >100 genes are shown for clarity of presentation and visualization, and the detailed information of all 129 MLGs is given in Table S2.

To explore detailed signatures of the gut microbiome in GDM patients and heathy controls, we constructed a non-redundant gene catalogue consisting of 4.34 million genes, which allowed an average reads mapping rate of 79.5% for sequenced samples. We identified 154 837 genes that displayed significant abundance differences between the 2 groups (Mann–Whitney U test, *q* < 0.05) (Fig. S1 shows the *P*-value distribution between GDM patients and healthy pregnant women for all genes tested). About 68% of these genes were clustered into 129 metagenomic linkage groups (MLGs) (Table S3), which allowed species-level description for the microbiome differences. The 71 MLGs enriched in GDM patients included *Parabacteroides distasonis, Klebsiella variicola, Catenibacterium mitsuokai, Coprococcus comes*, and *Citrobacter* spp., whereas the 58 MLGs enriched in healthy pregnant women included *Methanobrevibacter smithii, Alistipes* spp. (*A. shahii, A. senegalensis*), *Bifidobacterium* spp. (*B. animalis, B. pseudocatenulatum*), and *Eubacterium* spp. (*E. siraeum, E. eligens*). The GDM-enriched and control-enriched MLGs were highly positively interconnected within each group; however, few negative connections were found between the 2 groups (Fig. 2). Notably, GDM-enriched MLGs of *Enterobacteriaceae*, including *K. variicola, E. coli, Enterobacter cloacae*, and *Citrobacter* spp., were closely linked (correlation coefficients > 0.40 between each other), representing a cooperative promoting function of *Enterobacteriaceae* to GDM development. Of particular interest, we also observed that the relative abundance of *Enterobacteriaceae* was positively associated with pre-pregnancy body mass index (PBMI) (Fig. S2).

### Correlations between maternal blood glucose levels and gut microbiota

**Figure 3:**
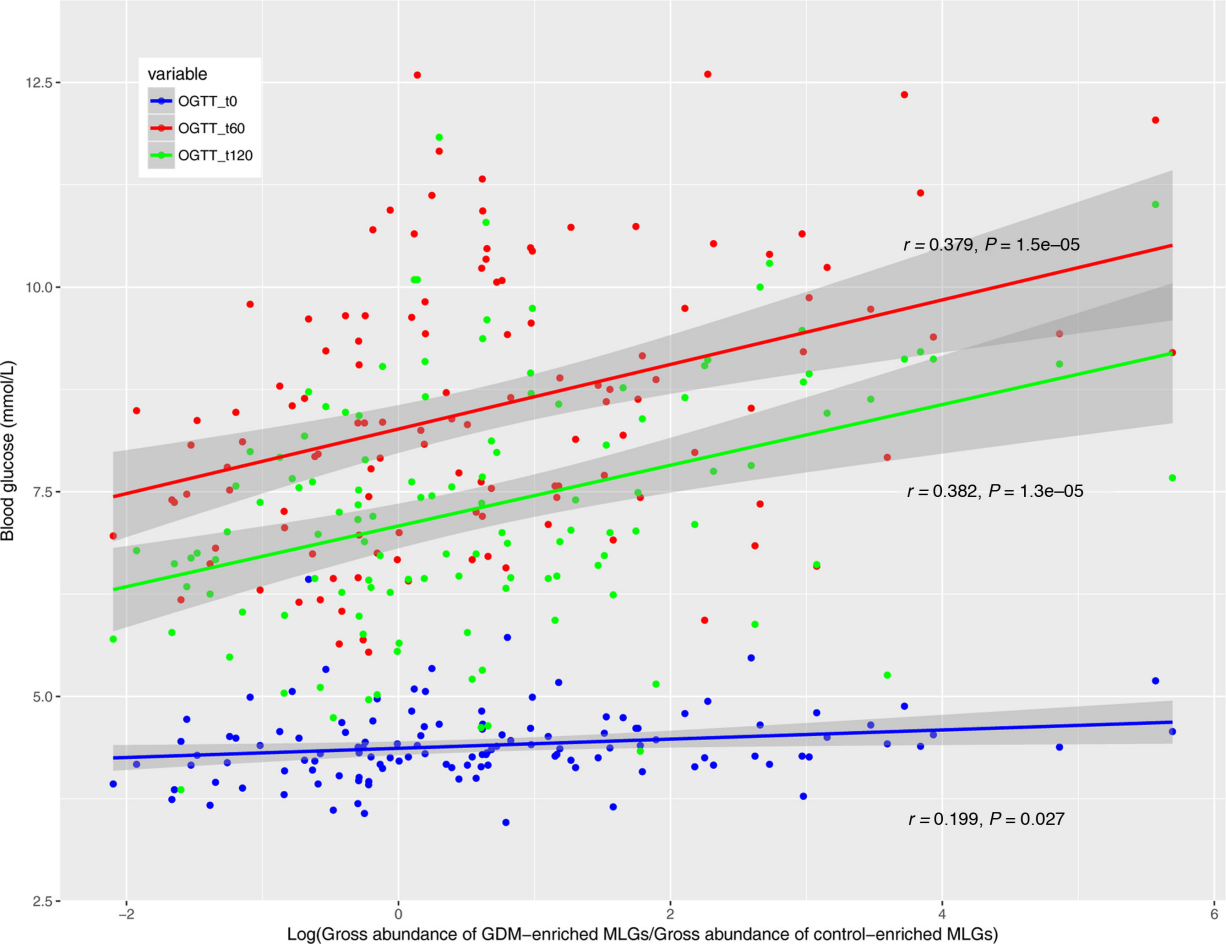
Association of gross abundance of GDM-enriched and control-enriched MLGs with blood glucose levels 0, 60, and 120 minutes after an oral glucose tolerance test. Scatter plots of all samples (including GDM patients and healthy controls) are shown with lines indicating linear fit.

**Figure 4:**
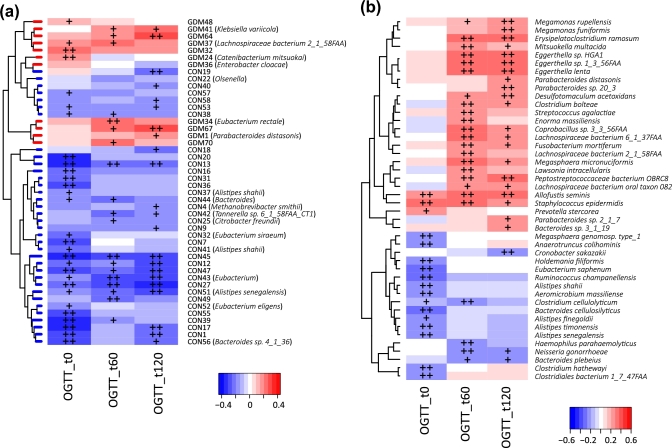
Correlation of blood glucose levels 0, 60, and 120 minutes after an oral glucose tolerance with MLGs (**a**) and species (**b**). Spearman's rank correlation coefficients and *P*-values for the correlations are shown. The plus sign denotes *P* < 0.05; double plus sign denotes *P* < 0.01. Only MLGs or species with average relative abundances greater than 0.001% and correlated (*P* < 0.05) with at least 1 index are shown for clarity.

In order to explore the potential clinical paths by which changes in the microbiome might lead to GDM, we investigated whether the MLGs can affect blood glucose tolerance. The ratios of the gross abundances of GDM-enriched MLGs to those of control-enriched MLGs were obviously positively correlated with blood glucose levels during the second trimester of pregnancy (Fig. 3), indicating that dysbiosis of the microbiome has a significant relationship with GDM status. Several GDM-enriched MLGs (e.g., GDM67, GDM64, *P. distasonis* [GDM1], *K. variicola* [GMD41], and *E. rectale* [GDM34]) were positively correlated with blood glucose levels, while most control-enriched MLGs were negatively correlated with blood glucose levels (Fig. 4a). At the species level, *Eggerthella* spp., *Megamonas* spp., *Allofustis seminis*, and several species in *Lachnospiraceae* and *Parabacteroides* were positively correlated with glucose tolerance, while several *Alistipes* spp. were negatively correlated with glucose tolerance (Fig. 4b).

### Functional characterization of the gut microbiota in GDM

**Figure 5:**
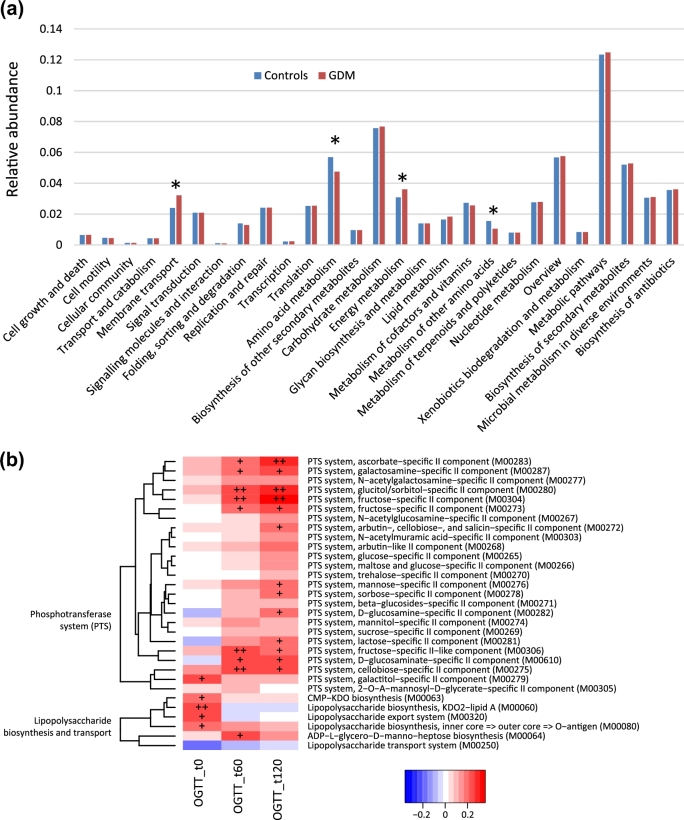
Association of microbial genetic functional pathway composition in GDM patients and healthy pregnant women. (**a**) Distributions of relative abundances of KEGG pathway categories in GDM patients and healthy controls. The asterisk denotes *q* < 0.05 (Mann–Whitney U test corrected by the Benjamini–Hochberg method). (**b**) Correlation of blood glucose levels 0, 60, and 120 minutes after an oral glucose tolerance test, with PTS system and LPS biosynthesis and transport system. Spearman's rank correlation coefficients and *P*-values for the correlations are shown. The plus sign denotes *P* < 0.05; double plus sign denotes *P* < 0.01.

Next, we utilized Kyoto Encyclopedia of Genes and Genomes (KEGG) pathway comparisons to explore potential differences in the functional composition of the microbiome of GDM patients versus controls. Although the functional composition of GDM patients and controls was highly similar (Fig. 5a), the microbiome of GDM patients showed a greater abundance in pathways of membrane transport and energy metabolism, while the microbiome of controls had higher abundance in amino acid metabolic pathways. We also found that the KEGG modules, including the phosphotransferase system (PTS) and lipopolysaccharide (LPS) biosynthesis and export systems, were associated with glucose tolerance levels (Fig. 5b).

### Gut microbiota–based prediction of GDM

**Figure 6:**
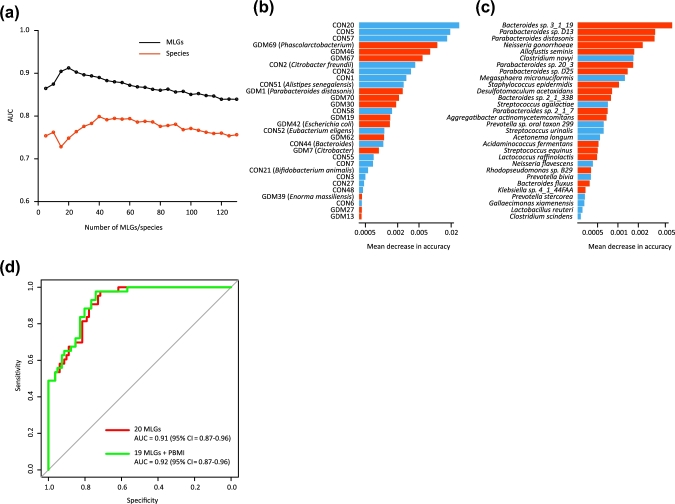
Classification of GDM status by the relative abundance of MLGs and species. (**a**) Classification performance of a random forest model using MLG or species abundance assessed by AUC. The performance was explored for different numbers of explanatory variables, ordered by importance. (**b, c**) The 30 most discriminant MLGs (b) and species (c) in the models classifying GDM and controls. The bar lengths in (b) and (c) indicate the importance of the variable, and the colors represent enrichment in GDM (red shades) or controls (blue shades). (**d**) ROC analysis for classification of GDM status by MLGs and PBMI.

Finally, we utilized random forest models to assess the predictive ability of MLGs and species abundance profiles for GDM status. We found that certain 20 MLGs provided the best discriminatory power, as indicated by the area under the receiver operating characteristic (ROC) curve (AUC) 0.91 (95% confidence interval [CI] = 0.87–0.96), which was higher than that achieved using species profiles with this model (the best AUC was 0.80; 95% CI = 0.73–0.86) using 40 species (Fig. 6a). The increased AUC for the MLG-based model may be due to the fact that MLGs furnish taxonomic and functional information for unknown or unanalyzable species. Bacterial species providing the highest discriminatory power were primarily members of the *Bacteroides* or *Parabacteroides* genera (Fig. 6b and c), consistent with our observation that *Parabacteroides* is the predominant genus accounting for differences in the gut microbiome between GDM patients and controls (Fig. 1b). Although PBMI is a predictor of GDM, it did not substantially improve the performance of MLGs (Fig. 6d; Fig. S3).

## Discussion

**Figure 7:**
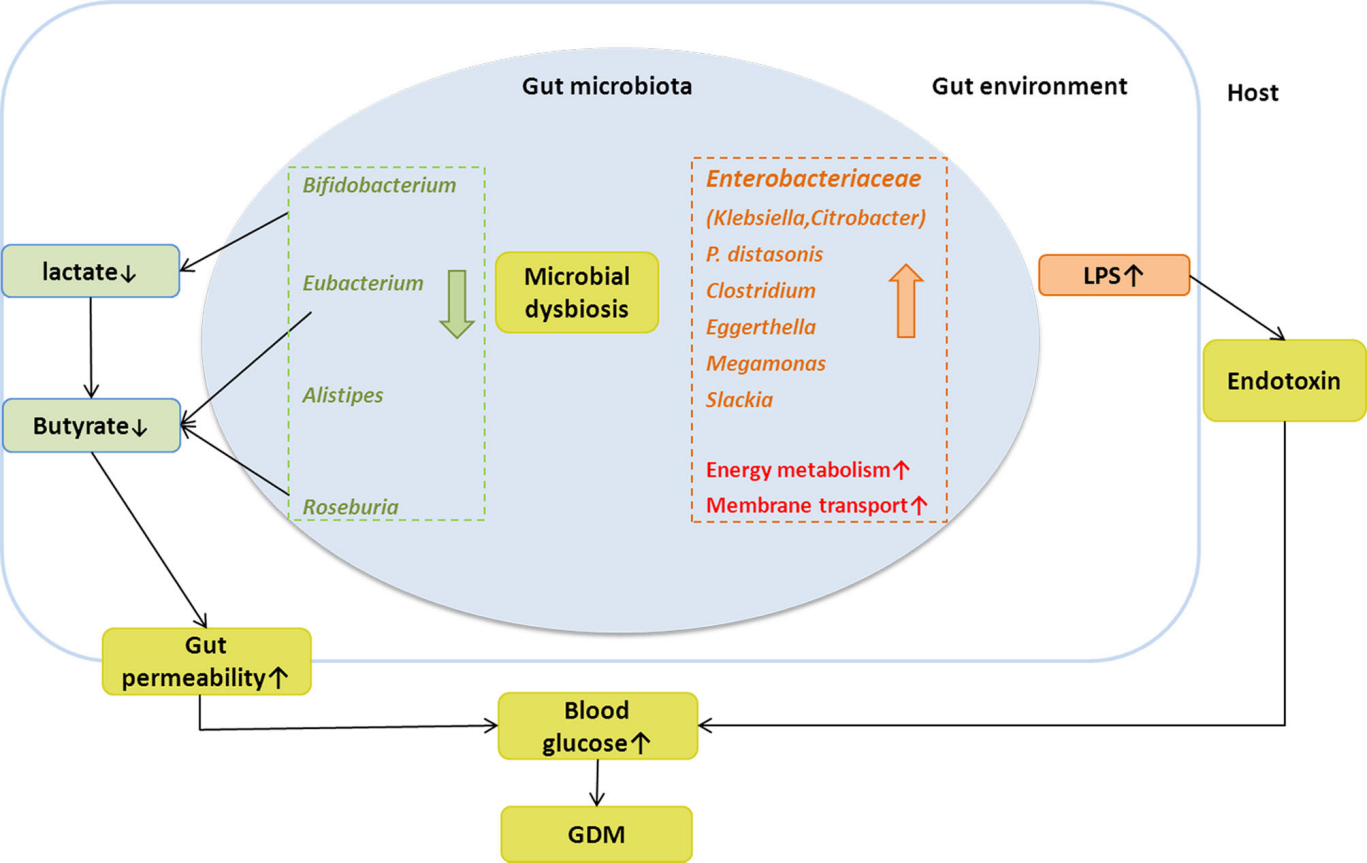
A schematic diagram showing the main bacteria and functions of the gut microbes that had a predicted GDM association. Red and orange columns and text denote enriched bacteria and their putative functions in GDM patients; green columns and text denote depleted bacteria and their putative functions in GDM patients.

In the present metagenomics study, we observed associations between gut microbiome and GDM status. Specifically, *Parabacteroides distasonis, Klebsiella variicola*, etc., were enriched in GDM patients, whereas *Methanobrevibacter smithii, Alistipes* spp., *Bifidobacterium* spp., and *Eubacterium* spp. were enriched in controls. The distribution of MLGs in GDM patients differed from that in the control group. Functional analysis showed a greater abundance of membrane transport, energy metabolism pathways, lipopolysaccharide, and phosphotransferase systems in the microbiome of GDM patients, while the microbiome of controls was enriched in the amino acid metabolic pathways (Fig. 7). To our knowledge, this is the first metagenomics study exploring the roles of microbiota in the development of GDM.

Previous studies have shown that the GDM-enriched bacteria observed in our study are involved in gut flora dysbiosis. For example, GDM-enriched *Bacteroides* spp. and *Parabacteroides distasonis* are considered opportunistic pathogens in infectious diseases, with potential for developing antimicrobial drug resistance [19]. The family *Enterobacteriaceae* also occurred with a higher relative abundance in GDM patients than in healthy controls, which indicates a status of gut flora dysbiosis that may lead to a series of chronic diseases, such as colitis [20], Crohn's disease, and acute cholecystitis [21]. Previous studies have shown that *Enterobacteriaceae* instigate inflammation to induce colitis [20] and that the endotoxin-producing bacterium *Enterobacter* contributed to the development of obesity in gnotobiotic mice [22].

The decreased microbes in GDM patients included *Bifidobacterium* spp. (including *B. pseudocatenulatum, B. animalis*, and 1 unclassified MLG), *Eubacterium* spp. (*E. siraeum, E. eligens*, and 2 unclassified *Eubacterium* MLGs), and *Roseburia* spp. (Tables S2 and S3). Similar findings were reported in previous studies on a variety of chronic diseases, including T2D [23], liver cirrhosis [24], Crohn's disease [25], and ulcerative colitis [26]. These bacteria can produce lactate or butyrate, which could regulate gut permeability and induce the gut inflammatory response that precedes the development of diabetes [27, 28].

Our data demonstrated that the ratio of gross abundances of the GDM-enriched to control-enriched MLGs was positively correlated with blood glucose tolerance levels, suggesting that microbiome dysbiosis might have a direct association with GDM pathophysiology. Functional analysis showed that the LPS biosynthesis and export systems were involved in the regulation of glucose levels. Previous studies have shown that higher systemic LPS levels were associated with low-grade chronic inflammation in obesity, metabolic syndrome, and T2D [8, 29, 30]. Based on current knowledge, the possible pathways linking LPS levels to glucose metabolism may include the increases in intestinal permeability, the changes in the relative amounts of gram-negative versus gram-positive bacteria, and a low-grade chronic inflammatory state. LPS is a bacterial cell wall component in gram-negative bacteria and can stimulate an inflammatory response [31, 32]. Gut microbiome dysbiosis can facilitate LPS entry into systemic circulation through increasing gut permeability, which leads to inflammation and metabolic dysfunction [33]. Our results were concordant with a previous report [23] that found that gut microbiota dysbiosis in T2D was characterized by a decrease in gram-positive butyrate, producing *Clostridium* species that lack LPS and an increase in gram-negative opportunistic pathogens including some *Bacteroidetes* and *Proteobacteria* species that contain LPS. The functional analysis in the present study found that membrane transport, energy metabolic, and PTS pathways were enriched in the GDM patients. PTS pathways are responsible for transporting glucose through outer and inner membranes and catalyzing the uptake of carbohydrates. The increased relative abundance of these pathways may indicate that the gut environment of a GDM status may stimulate accelerated bacterial usage of glucose as energy.

There were several limitations in our study. First, the sample size is relatively small. Second, we only analyzed 1 stool sample per participant, which was collected in the second trimester of pregnancy. It is well known that immune and metabolic changes occur throughout pregnancy and that the gut microbiota shifts from the first to third trimesters [16]. In the present study, we are unable to clarify the causal relationship between the microbiome and the development of GDM due to the cross-sectional design. Consequently, data at multiple time points are needed to provide further insights into their dynamic relationship. Third, we did not have information on several factors; e.g., lifestyle and diet may further affect both blood glucose levels and gut microbiota composition. In order to more confirm the associations observed in the current study, a large prospective cohort investigation with analysis of other potentially significant variables will be necessary. Additionally, due to the lack of serum samples, we could not measure LPS levels and describe the real endotoxemia level of the patients.

In summary, this is the first study to demonstrate an association between the gut microbiota dysbiosis, functional changes, and GDM. Our findings contribute to the understanding of GDM pathophysiology and may have important implications for identifying patients at risk for the development of GDM.

### Potential implications

The gut microbiome can be considered both an endocrine and a metabolic organ, the dysfunction of which plays important roles in disease development. During gestation, profound hormonal, immunological, and metabolic changes take place [34–36]. Our findings suggest that gut microbiota in pregnant women are sensitive to subtle changes in metabolism and increases in blood glucose levels. When taken together with results from previous studies on T2D [23], our findings suggest that gut microbiota may be a potential predictor of T2D after pregnancy. Furthermore, data from our cohort indicate that women diagnosed with GDM also suffered from moderate gut bacterial dysbiosis and functional dysbiosis that was not restricted to certain microbial species. Although causality has not been demonstrated, it raises the possibility that susceptibility of postpartum metabolic (e.g., T2D) and immune dysfunction might be modified by reconditioning of gut microbiota. Given that the gut microflora can be modified by diet, altering test the composition of gut microbiota in pregnant women may improve diabetes-related outcomes. Future studies should explore how gut bacterial dysbiosis could be improved and evaluate the efficacy of potential interventions, such as probiotics and dietary manipulation, among pregnant women.

## Methods

### Study population and sampling

As part of the Born in Guangzhou Cohort Study (BIGCS) [37], fecal samples were obtained from 298 pregnant women during their second trimester in Guangzhou Women and Children's Medical Center (GWCMC) between 1 August 2012 and 31 August 2013. The inclusion criteria of the current study were as follows: (i) without diseases that might affect glucose metabolism or microbiome composition such as pre-pregnancy diabetes, hypertension, thyroid disorders, asthma, lipid metabolic disorders, inflammatory bowel disease, irritable bowel syndrome, and celiac disease; (ii) had not received any antibiotic treatment 1 month before sample collection; (iii) had not taken probiotics 2 weeks before sample collection. Of the 287 eligible women, 43 had a diagnosis of GDM and were included in the present study as the case group, and 81 non-GDM women were randomly selected as the control group. Basic characteristics of the 124 pregnant women included in the study are summarized in Table S1. Compared to non-GDM women, women with GDM were more likely to be older and multiparous and have higher pre-pregnant weight, pre-pregnancy body mass index (BMI), gestational weight gain during pregnancy, and premature delivery incidence. Fecal samples were frozen in –20°C freezers immediately (within 30 minutes) and transferred to –80°C freezers within 24 hours after collection.

This study received approval from the Ethics Committee of GWCMC, and written informed consent was obtained from all participating pregnant women. Participants underwent a standard 2-hour 75-g oral glucose tolerance test (OGTT) between 21 and 29 weeks’ gestation by collection of 2-ml blood samples when fasting, at 1 hour, and at 2 hours after a 75-g glucose load, using NaF/EDTA tubes. After centrifugation, plasma glucose was measured by a hexokinase method using a Beckman Coulter AU5800 automatic analyzer (Beckman Coulter, Brea, CA, USA). The laboratory previously achieved ISO15189 certification by China National Accreditation Service for Conformity Assessment. GDM was defined using the Chinese diagnostic criteria [38], which is in agreement with the one-step approach endorsed by the American Diabetes Association [39]. Pregnant women were diagnosed as having GDM if 1 or more of the following glucose levels were elevated: fasting ≥5.1 mmol/L, 1 hour ≥10.0 mmol/L, and 2 hours ≥8.5 mmol/L [38]. None of these women was treated with insulin or glyburide. Maternal age, pre-pregnancy weight, and pre-pregnancy height were extracted from clinical records of the Hospital Information Systems used in GWCMC. Pre-pregnancy body mass index was calculated from height and weight information.

### DNA extraction and metagenomic sequencing

Total bacterial DNA was extracted from about 180–200 mg of feces using Qiagen QIAamp DNA Stool Mini Kit (Qiagen) following the manufacturer's instructions [40]. Extracted DNA of each sample was kept frozen at –20°C until used. Illumina HiSeq 2000 was used to sequence the samples. We constructed a paired-end library with insert size of 350 base pairs for every sample, and sequenced with a 100-bp read length from each end. Illumina sequencing reads for fecal samples from pregnant women were independently processed for quality control using the FASTAX Toolkit (FASTAX Toolkit, RRID:SCR_015042) [41]. The following criteria were used for quality control: (i) reads were removed if they contained more than 3 N bases or more than 50 bases with low quality (<Q20); (ii) reads were trimmed in the end with low quality (<Q20) or assigned as N. The remaining reads were then mapped to the human genome using SOAPalinger2 (SOAPaligner/soap2, RRID:SCR_005503) [42] to remove contaminating human DNA. After QC, an average of 1.9% of low-quality or human genome reads were removed for the 124 samples.

### *De novo* assembly, gene calling, and gene catalogue construction

To determine the best assembling method for the obtained high-quality Illumina sequencing reads, we compared the performance of 2 assemblers, SOAPdenovo v. 2.04 (SOAPdenovo2, RRID:SCR_014986; as previously used in the MetaHIT and IGC projects) [43, 44] and IDBA-UD v. 1.1.1 (a *de novo* assembler for metagenomic sequences) [45]. For the SOAPdenovo, we tested the k-mer length, ranging from 23 bp to 123 bp by 10-bp step for each sample, and selected the assembled contig set with longest N50 length. For the IDBA-UD, the parameters “–mink 21 –maxk 81 –step 20 –pre_correction” were used. For most samples, IDBA-UD obtained a better assembled contig set than SOAPdenovo. This could be attributable to the relative efficiency of IDBA-UD in assembling bacterial genomes within regions of highly uneven depth in metagenomic samples. As a result, we obtained an average of 197.9 ± 50.3 Mbp (mean ± SD) contig sets for each pregnant women sample, with N50 length of 8.8 ± 3.9 kbp. Unassembled reads from these samples were pooled and re-assembled by using IDBA-UD for further analysis.

Genes were predicted by MetaGeneMark [46] based on parameter exploration by the MOCAT pipeline (MOCAT, RRID:SCR_011943) [47]. A non-redundant gene catalogue of pregnant women samples was constructed using CD-HIT (CD-HIT, RRID:SCR_007105) [48], through which genes with >90% overlap and >95% nucleic acid similarity (no gap allowed) were removed as redundancies. A pregnant women gene catalogue containing 4 344 984 non-redundant genes was generated for fecal samples collected from these 124 pregnant women. This gene catalogue was further combined with the previous integrated gene catalogue (IGC) [44] by removing redundancies (2 621 398 genes) in the same manner as above. In the end, 39.6% (1 723 586) of the genes in the pregnant women gene catalogue were identified as novel.

### Quantification of metagenomic genes

The abundance of genes in the combined non-redundant gene catalogue (combining the pregnant women gene catalogue and IGC) was quantified as a relative abundance of reads. First, high-quality reads from each sample were aligned against the gene catalogue using SOAP2.21 [42], with thresholds that allowed a maximum of 2 mismatches in the initial 32-bp seed sequence and 90% similarity over the whole reads. Only 2 types of alignments were accepted: (i) the entire paired-end read can be mapped onto a gene with the correct insert size; (ii) 1 end of the paired-end read can be mapped onto the end of a gene only if the other end of the read was mapped outside the genic region. The relative abundance of a gene in a sample was estimated by dividing the number of reads that uniquely mapped to that gene by the length of the gene region and by the total number of reads from the sample that uniquely mapped to any gene in the catalogue. The resulting set of gene relative abundances of a sample was its gene profile.

### Richness

We used the gene count and Shannon index to represent the richness and evenness of the gut microbiota for each sample. As defined previously [5], the gene counts of a metagenomic sample were calculated based on their reads mapping number on the non-redundant gene catalogue. To eliminate the influence of sequencing depth fluctuation, an equal number of 11 million reads for all samples was randomly extracted for mapping, and then the mean number of genes over 30 random drawings was generated. The Shannon index (within sample diversity) was calculated as previously described [23].

### Taxonomical and functional analyses

#### Taxonomical classification of genes

Reference microbial genomes were downloaded from the NCBI-genome database (v. May 2015), which included 8953 bacterial/archaea genomes (of which 2785 genomes were complete and 6168 were draft genomes) and 4400 viral genomes. Genes from the non-redundant gene catalogue were aligned to reference genomes using BLASTN (BLASTN, RRID:SCR_001598) with parameters “-word_size 16 -evalue 1e-10 -max_target_seqs 5000.” At least 70% alignment coverage of each gene was needed. Based on the parameter exploration of sequence similarity across phylogenetic ranks [49], we used 85% identity as the threshold for genus assignment, and 65% for phylum assignment.

#### Functional annotation of genes

The Kyoto Encyclopedia of Genes and Genomes (KEGG orthologous, v. April 2015; KEGG, RRID:SCR_012773) and evolutionary genealogy of genes: Non-supervised Orthologous Groups (eggNOG, v. 4; eggNOG, RRID:SCR_002456) databases were used for functional annotation of genes. Translated amino acid sequences of genes were searched against these databases using USEARCH v. 8.0.1616 (evalue < 1e-5, query_cov > 0.70) [50] with a minimum similarity of 30%. Each protein was assigned a KEGG orthologue (KO) or an eggNOG orthologue group (OG) based on the best-hit gene in the database. Using this approach, 43.6% and 71.9% of the genes in the combined gene catalogue could be assigned a KO or an OG, respectively. As a final step, the abundance profiles of KEGG and eggNOG were calculated by summing up the relative abundance of genes annotated to a feature.

### Metagenome-wide association study 

We used the metagenome-wide association study (MGWAS) methodology to identify gene markers that showed significant abundance differences between the GDM and control individuals. The MGWAS was performed using methodology developed by Qin et al. [23]. Briefly, gene relative abundance profiles were initially adjusted for population stratifications using the modified EIGENSTRAT method [51], which allows the use of covariance matrices estimated from abundance levels instead of genotypes. Then, a 2-tailed Mann–Whitney U test was performed in the adjusted gene profiles, and the Benjamin–Hochberg procedure [52] was subsequently used to correct the *P*-values to generate the false discovery rate (FDR, known as “*q*-value”) for each gene.

### Metagenomic linkage group analysis

Co-abundance genes were clustered into MLGs based on the previously described methodology [23]. Taxonomic assignment and abundance profiling of the MLGs were performed according to the taxonomy and relative abundance of their constituent genes, as previously described [23]. Briefly, assignment to a species requires 90% of genes in an MLG to align with the species’ genome with 95% identity and 70% overlap of query. Assigning an MLG to a genus requires 80% of its genes to align with a genome with 85% identity in both DNA and protein sequences. MLGs were further interconnected according to Spearman's correlation coefficient (ρ > 0.4 or ρ < −0.4) between their abundances in all GMD and control samples, and the co-occurrence network of MLGs was visualized by Cytoscape 3.0.2 (Cytoscape, RRID:SCR_003032) [53]. The direction of enrichment was determined by the Mann–Whitney U test (*P* < 0.05).

### Statistical analysis

Statistical analysis was implemented using the R platform. Distance-based redundancy analysis was performed using the “vegan” package [54] based on the Bray–Curtis distances on normalized taxa relative abundance matrices, then visualized using the “ggplot2” package. Permutational multivariate analysis of variance was performed using the “vegan” package, and the permuted *P*-value was obtained by 10 000 permutations.

The Random Forest model has been shown [6] to be a suitable model for exploiting metagenomic data. Random Forest models were trained using the “randomForest” package (default parameters and 10 000 trees) to identify GDM status in a subset of GDM patients and control group by using the abundance profiles of species and MLGs. Performance of the predictive model was evaluated with cross-validation error. Variable importance by mean decrease in accuracy was calculated for the Random Forest models using the full set of species or MLGs. Based on the rank of variables by importance, concise models were constructed that contained only the most important variables.

Receiver operator characteristic analysis was performed using the “pROC” package; we then computed the 95% confidence interval of the AUC with 10 000 bootstrap replicates to assess the variability of the measure. Rarefaction analysis was performed to assess the gene richness of metagenomic samples, implemented by in-house Perl scripts.

## Availability of supporting data and materials

All raw sequencing data have been deposited in the EBI Sequence Read Archive under accession number ERP020710. Further supporting data is available in the *GigaScience* repository, *Giga*DB [55].

## Additional files

Supplemental File Figure S1. Density histogram showing the *P*-value distribution between GDM patients and healthy pregnant women for all genes tested. The horizontal line represents the expected distribution of *P*-values, and the π0 value indicates the proportion of genes under the null hypothesis.

Supplemental File Figure S2. Correlation between *Enterobacteriaceae* relative abundance and PBMI. Scatter plots of samples are shown with lines indicating linear fit.

Supplemental File Figure S3. Classification of GDM status by abundance of MLGs and PBMI. The 30 most discriminant MLGs or PBMI in the models for classifying GDM and controls. The bar lengths indicate the importance of the variable, and colors represent enrichment in GDM (red shades) or controls (blue shades).

Supplemental File Table S1: Characteristics of the study participants (mean ± SD [range] or N/N(%/%)).

Supplemental File Table S2: Bacterial species that differed significantly between 2 cohorts.

Supplemental File Table S3: Detailed information of 129 GDM-associated MLGs.

## Abbreviations

AUC: area under the curve; bp: base pairs; BMI: body mass index; CI: confidence interval; GDM: gestational diabetes mellitus; IGC: integrated gene catalogue; KEGG: Kyoto Encyclopedia of Genes and Genomes; KO: KEGG group; LPS: lipopolysaccharide; MGWAS: metagenome-wide association study; MLGs: metagenome linkage groups; PBMI: pre-pregnancy body mass index; PTS: phosphotransferase system; OG: orthologue group; OGTT: oral glucose tolerance test; ROC: receiver operating characteristic curve; T2D: type 2 diabetes.

## Consent for publication—human data

This study was approved by both the institutional review board and the ethics committee at GWCMC. All protocols were conducted in compliance with the Declaration of Helsinki, and explicit informed consent was obtained from all participants.

## Competing interests

The authors declare that they have no competing interests.

## Funding

This study is supported by the National Natural Science Foundation of China (81673181), Guangzhou Science and Technology Bureau, Guangzhou, China (201508030037), and Shenzhen Municipal Government of China (CXB201108250098A and JSGG20160229172752028). The sponsors had no role in design or conduct of the study; the collection, management, analysis, or interpretation of the data; the preparation, review, or approval of the manuscript; or the decision to submit the manuscript for publication.

## Author contributions

X.Q. and H.X. conceived and supervised the project. Y.K., M.Y., J.H., J.L.,* N.C., W.X., S.S., L.Q., Y.W., C.H., Q.C., W.L., and Y.W. oversaw sample collection and provided phenotypic information. Y.K., J.L.,* and S.L. analyzed the data and drafted the manuscript. X.Q., H.D., J.L., and C.P. performed substantial revision of the manuscript. All authors critically revised the manuscript and approved the final version. J.L.* represents Jin-Hua Lu.

## Supplementary Material

GIGA-D-16-00167_Original-Submission.pdf

GIGA-D-16-00167_Revision-1.pdf

GIGA-D-16-00167_Revision-2.pdf

GIGA-D-16-00167_Revision-3.pdf

Response-to-Reviewer-Comments_Original-Submission.pdf

Response-to-Reviewer-Comments_Revision-1.pdf

Response-to-Reviewer-Comments_Revision-2.pdf

Reviewer-1-Report-(Original-Submission).pdf

Reviewer-1-Report-(Revision-1).pdf

Reviewer-2-Report-(Original-Submission).pdf

Reviewer-2-Report-(Revision-1).pdf

Reviewer-2-Report-(Revision-2).pdf

Reviewer-2_Original-Submission-(Attachment).pdf

Additional Files
