# Supplementary material for: Connections between the human gut microbiome and gestational diabetes mellitus
Source: Gigascience. 2017 Jul 31;6(8):1–12. doi: 10.1093/gigascience/gix058 (PMC5597849; doi:10.1093/gigascience/gix058)
Supplement: GIGA-D-16-00167_Original-Submission.pdf [file gix058_GIGA-D-16-00167_Original-Submission.pdf]

Manuscript
Click here to download Manuscript maintext-20161215-1.docx

Connections between human gut microbiome and gestational diabetes mellitus

Yong Guo[1,2,†], Ya-Shu Kuang[1,†], Sheng-Hui Li[1,†], Jun-Hua Li[3,4], Ming-Yang Yuan[1,2], Jian-Rong He[1,2], Jin-Hua Lu[1,2], Nian-Nian Chen[1,2], Wan-Qing Xiao[1,2], Song-Ying Shen[1,2], Lan Qiu[1,2], Ying-Fang Wu[1,2], Cui-Yue Hu[1,2], Yan-Yan Wu[1,2], Wei-Dong Li[1,2], Qiao-Zhu Chen[5], Hong-Wen Deng[1,6], Christopher J Papasian[7], Hui-Min Xia[1,8*], Xiu Qiu[1,2 *]

[1] Division of Birth Cohort Study, Guangzhou Women and Children's Medical Center, Guangzhou Medical University, Guangzhou 510623, China

[2] Department of Women and Children's Health, Guangzhou Women and Children's Medical Center, Guangzhou Medical University, Guangzhou 510623, China

[3] BGI-Shenzhen, Shenzhen 518083, China

[4] BGI Hong Kong Research Institute, Hong Kong, China

[5] Department of Obstetrics and Gynecology, Guangzhou Women and Children's Medical Center, Guangzhou Medical University, Guangzhou 510623, China

[6] Center of Bioinformatics and Genomics, Department of Biostatistics and Bioinformatics, Tulane School of Public Health and Tropic Medicine, USA

[7] Department of Basic Medical Science, School of Medicine, University of Missouri – Kansas City, 2411 Holmes St., Kansas City, MO 64108

[8] Department of Neonatal Surgery, Guangzhou Women and Children's Medical Center, Guangzhou Medical University, Guangzhou 510623, China

E-Mails:
geyong084@164.com (YG); kuangyashu@126.com (YSK); lishenghui1005@gmail.com (SHL); lijunhua@genomics.cn (JHL); ymyeasy@126.com (MYY); hjr0703@163.com (JRH); lujinhua10@gmail.com (JHL); cnnfezx@163.com (NNC); 15915711316@163.com (WQX); shsy_22@163.com (SYS); 38904442@qq.com (LQ); mywachy@163.com (YFW); bighcy@163.com (CYH); 153050569@qq.com (YYW); liweidong30303@163.com (WDL); bigcqz@163.com (QZC); hdeng2@tulane.edu (HWD); PapasianC@umkc.edu (CJP); huimin.xia876001@gmail.com (HMX); qxiu0161@163.com (XQ)

[†] The authors contributed equally to this work.

[*] Correspondence to:
Xiu Qiu, Division of Birth Cohort Study, Guangzhou Women and Children's Medical Center, Guangzhou Medical University, 9 Jinsui Road, Guangzhou 510623, China; Phone: 86 2038367162; Fax: 86 2038367162; E-mail: qxiu0161@163.com.
Hui-Min Xia, Division of Birth Cohort Study, Guangzhou Women and Children's Medical Center, Guangzhou Medical University, 9 Jinsui Road, Guangzhou 510623, China; Phone: 86 2038076019; Fax: 86 2038076019; E-mail: huimin.xia876001@gmail.com.

Abstract

Background

Human gut microbiome can modulate metabolic health and affect insulin resistance, and may play an important role in the etiology of gestational diabetes mellitus (GDM). Here, we compared the gut microbial composition of 43 GDM patients and 81 healthy pregnant women via whole-metagenome shotgun sequencing of their fecal samples collecting at 21-29 weeks, to explore associations between GDM and the composition of microbial taxonomic units and functional genes.

Results

Metagenome-wide association study (MGWAS) identified 154,837 genes, which enabled to cluster into 129 metagenome linkage groups (MLGs) for species description, with significant abundance differences between two cohorts. *Parabacteroides distasonis*, *Klebsiella variicola*, etc., were enriched in GDM patients, whereas *Methanobrevibacter smithii*, *Alistipes* spp., *Bifidobacterium* spp. and *Eubacterium* spp. were enriched in controls. The GDM-associated species showed correlations with maternal blood glucose, indicating a potential relationship between gut microbes and blood glucose tolerance. We further evaluated the performance of gut microbiota as biomarker to identify GDM status with a Random Forest model and demonstrated that fecal MLGs may offer new indicators for prognosis of GDM.

Conclusions

Our study discovered novel relationships between gut microbiome and GDM status, and suggested that changes in microbial composition may potentially be used to identify individuals at risk for GDM.

Background

The increasing prevalence of gestational diabetes mellitus (GDM), and its subsequent health outcomes, are a significant public health concern and a major challenge for obstetric practice [1]. GDM represents a heterogeneous group of metabolic disorders [2] which affects 3-14% of pregnancies, and 20-50% of these affected women are expected to develop type 2 diabetes (T2D) within 5 years [3, 4]. Emerging evidence has revealed a link between the gut microbiome and

human metabolic health [5, 6], leading us to hypothesize that the gut microbiome may impact gestational metabolism and development of GDM.

Microbial dysbiosis in the human gut may be an important environmental risk factor for abnormal host metabolism, as recently exemplified in the studies of obesity and T2D (reviewed by Karlsson, et. al)[7]. A study using experimental animal model revealed reduced numbers of Bifidobacteria led to enhanced endogenous lipopolysaccharide production, endotoxemia, and associated obesity and insulin resistance [8]. In humans, excessive weight gain and obesity in pregnancy resulted in deteriorated glucose tolerance and increased risk of GDM [9, 10]. *Prevotella copri* and *Bacteroides vulgatus* have been identified as the main species driving the association between biosynthesis of branched-chain amino acids, insulin resistance, and glucose intolerance [11], and *Bacteroides* spp. and *Staphylococcus aureus* are significantly more abundant in overweight women than in normal-weight women [12].

While the majority of previous studies have focused on associations between intestinal microbiota and obese states or T2D [6, 13-15], some recent studies have sought to characterize microbiota changes during pregnancy, with the goal of providing novel insights into the relationship between microbiota changes during pregnancy and potential metabolic consequences [16]. Studies based on sequencing of 16S ribosomal RNA have revealed novel relationships between gut microbiome composition and the metabolic hormonal environment in overweight and obese pregnant women in early gestation [17]. Koren et al. found that maternal gut microbiota changed from first to third trimesters, with a decline in butyrate-producing bacteria and increased Bifidobacteria, *Proteobacteria*, and lactic-acid producing bacteria [16]. Further, transplants of fecal material obtained during different trimesters were sufficient to confer different phenotypes in mouse models, with third-trimester fecal transplants leading to increased adiposity and inflammation [16]. These studies suggest that pregnancy is associated with major shifts in the gut microbiome which may play an important role in observed increases in gestational inflammation, thereby potentially contributing to development of GDM. However, studies focusing on changes in the gut microbiome during pregnancy and development of GDM have not been reported so far.

Metagenomic shotgun sequencing, in which the full complement of genes present in the microbiome are sequenced, can furnish information about the relative abundance of genes in functional pathways and at all taxonomical levels [18]. In this study, we used whole-metagenome

shotgun sequencing analyses of the gut microbiome during pregnancy to explore associations between GDM and the composition and abundance of microbial taxonomic units and functional genes. The objective was to obtain a comprehensive understanding of the gut microbiome's role in the etiopathogenesis of GDM.

Data description

We obtained the fecal samples from 124 pregnant women, including 43 GDM patients and 81 healthy control individuals, during their second trimester in Guangzhou Women and Children's Medical Center (GWCMC). Whole-metagenome shotgun sequencing of the samples were preformed based on the Illumina HiSeq2000 platform in BGI-Shenzhen, China. We constructed a paired-end library with insert size of 350 base pairs (bp) for every sample, and sequenced with 100bp read length from each end. Sequencing reads for fecal samples were independently processed for quality controlling and host sequences removing based on an in-house pipeline (see Methods), and totaling 795 Gbp high quality metagenomic data (average per sample, 6.4 Gbp) were finally generated for further analysis. We performed *de novo* assembly and gene calling for data of each sample, and constructed a non-redundant gene catalogue of all pregnant women samples containing 4,344,984 genes. This gene catalogue provided a suitable reference for metagenomic gene quantification, microbial diverisity analysis, and metagenome-wide association study for the pregnant women samples.

Results

Comparison of the gut microbiota between GDM patients and healthy pregnant women

First, we explored potential differences in the gut microbiome between 43 GDM patients and 81 healthy pregnant women. In order to perform this analysis, we obtained 795.3 Gb of high-quality data (6.4 ± 1.3 Gb per sample, Table S1) via metagenomic shotgun sequencing of their fecal samples. We aligned the sequencing reads (43.8%) against available microbial genomes from the National Center for Biotechnology Information and generated taxonomic composition for all samples at the taxonomic levels of phylum, class, order, family, genus and species. Multivariate analysis based on Bray–Curtis distances between microbial genera revealed significant differences between GDM patients and healthy controls (Figure 1a). We then preformed the Mann–Whitney U

test to identify phylogenetic differences between GDM patients and healthy controls. No significant differences were found at the phylum and class levels, however, the order *Clostridiales* was enriched in healthy controls. At the genus level, GDM patients had a significantly higher abundance of *Parabacteroides*, *Megamonas* and *Phascolarctobacterium*, while healthy controls were significantly enriched for *Ruminiclostridium*, *Roseburia*, *Eggerthella*, *Fusobacterium*, *Haemophilus*, *Mitsukella*, and *Aggregatibacter* (Figure 1b). We also found a number of bacterial species that differed significantly between GDM patients and healthy controls, consistent with the genus level observations (Table S2). These findings suggest dysbiosis of the gut microbiota based on GDM status.

Identification of GDM-associated markers from gut microbiome

To explore detailed signatures of the gut microbiome in GDM patients and heathy controls, we constructed a non-redundant gene catalogue consisting of 4.34 million genes, which allowed an average reads mapping rate of 79.5% for sequenced samples. We identified 154,837 genes that displayed significant abundance differences between the two groups (Mann-Whitney U test, $q<0.05$). (Figure S1 shows the P-value distribution between GDM patients and healthy pregnant women for all genes tested). ~68% of these genes were clustered into 129 MLGs (Table S3), which allowed species level description for the microbiome differences. The 71 MLGs enriched in GDM patients included *Parabacteroides distasonis*, *Klebsiella variicola*, *Catenibacterium mitsuokai*, *Coprococcus comes* and *Citrobacter spp.*, whereas the 58 MLGs enriched in healthy pregnant women included *Methanobrevibacter smithii*, *Alistipes spp.* (*A. shahii, A. senegalensis*), *Bifidobacterium spp.* (*B. animalis*, *B. pseudocatenulatum*) and *Eubacterium spp.* (*E. siraeum*, *E. eligens*). The GDM-enriched and control enriched MLGs were highly positively interconnected within each group; however, only a few negative connections was found between the two groups (Figure 2). Notably, GDM-enriched MLGs of *Enterobacteriaceae*, including *K. variicola*, *E. coli*, *Enterobacter cloacae* and *Citrobacter spp.*, were closely linked (correlation coefficients >0.40 between each other), representing a cooperative promoting function of *Enterobacteriaceae* to GDM development. Of particular interest, we also observed that the relative abundance of *Enterobacteriaceae* was positively associated with pro-gestational body mass index (PBMI, Figure S2).

Correlations between maternal blood glucose and gut microbiota

In order to explore the potential clinical paths by which changes in the microbiome might lead to GDM, we investigated whether the MLGs can affect blood glucose tolerance. The gross abundance of GDM-enriched and control-enriched MLGs were obviously associated with the blood glucose level during the second trimester of pregnancy (as measured by OGTT, Figure 3). Several GDM-enriched MLGs [e.g. GDM67, GDM64, *P. distasonis* (GDM1), *K. variicola* (GMD41) and *E. rectale* (GDM34)], were positively correlated with blood glucose levels, while a large proportion of control-enriched MLGs were negatively correlated the blood glucose levels (Figure 4a). At the species level, *Eggerthella spp.*, *Megamonas spp.*, *Allofustis seminis* and several *Lachnospiraceae* and *Parabacteroides* species, were positively correlated with glucose tolerance, while several *Alistipes spp.*, among others, were negatively correlated with glucose tolerance (Figure 4b).

Functional characterization of gut microbiota in GDM

Next, we utilized KEGG pathway comparisons to explore potential differences in the functional composition of microbiomes of GDM patients vs. controls. Although the functional composition of GDM patients and control subjects were highly similar (Figure 5a), the microbiome of GDM patients showed a greater abundance of membrane transport and energy metabolism pathways, while the microbiome of control subjects was enriched in the function of amino acid metabolic pathways. We also found that KEGG modules involving the phosphotransferase system (PTS, a major component of membrane transport) and lipopolysaccharide (LPS, a major component of the outer membrane of Gram-negative bacteria that induces a profound inflammatory response) biosynthesis and export systems, were associated with glucose tolerance levels (Figure 5b). These findings are consistent with those of a previous study [19] that reported an increase in microbial functions for membrane transport and LPS metabolism in the microbiome of patients with type 2 diabetes.

Gut microbiota-based classification of GDM

Finally, we utilized random forest models to assess the ability of MLGs and species abundance

profiles to predict GDM status. We found that 20 MLGs provided the best discriminatory power, as indicated by the area under the ROC curve (AUC) 0.91 (95% CI 0.87-0.96); this was higher than that achieved using species profiles with this model (the best AUC was 0.80; 95% CI 0.73-0.86) using 40 species (Figure 6a). The increased AUC for the MLG-based model may be due to the fact that MLGs furnish taxonomic and functional information for unknown or unanalyzable species. Bacterial species providing the highest discriminatory power were primarily members of the *Bacteroides* or *Parabacteroides* genera (Figure 6b-c), consistent with our earlier observation that *Parabacteroides* is the predominant genus accounting for differences in the gut microbiome between GDM patients and control subjects (Figure 1b). When age and PBMI of pregnant women were included along with the 129 MLGs, PBMI was selected as a marker together with the highest discriminatory MLGs, but the performance of the model did not obviously improve (Figure S3 and Figure 6d). Therefore, fecal MLGs, by themselves, may provide a mechanism and biomarkers for early detection of GDM or, potentially, for identifying risk of developing GDM.

Discussion

To identify and understand alterations in the gut microbiome associated with GDM, we characterized the genic, taxonomic, and functional repertoire of microbiomes of 43 GDM patients and 81 healthy pregnant women. To our knowledge, this is the first metagenomics study on stools of GDM patients, revealing significant dysbiosis, taxonomic shifts and functional changes in their microbiome as compared with healthy pregnant women.

Our study furnished a powerful set of microbial markers for GDM prediction, which achieved an AUC of 0.91, for identifying GDM status based on 20 species-level MLGs. The discriminatory power of this set of markers was higher than prediction models based on genomic markers identified by genome-wide association studies (GWAS) (AUC 0.5-0.7) [20]. Thus, as demonstrated in the current study, analysis of fecal microbiota could be used for the early diagnosis of GDM or, potentially, for identifying individuals at risk for developing GDM. Future systematic assessment of the key species and gene markers identified here will be required to further develop this tool.

Enterobacteriaceae, a bacterial family that contains many commensals as well as human

pathogens that causes pneumonia, diarrhea and urinary tract infections, occurred with higher relative abundance in GDM patients than in healthy controls. Overgrowth of *Enterobacteriaceae* usually indicates a status of gut flora dysbiosis, which may lead to a series of chronic diseases, such as colitis [21], Crohn's disease and acute cholecystitis [22]. In this study, the GDM-enriched *Enterobacteriaceae* species include *Klebsiella variicola, Escherichia coli*, *Enterobacter cloacea* and three Citrobacter MLGs (Table S3). Based on these findings, the association between *Enterobacteriaceae* and GDM is intriguing, but of uncertain pathogenic significance. This intriguing observation warrants further studies.

In the current study, microbes found to be enriched in the gut of control subjects included *Methanobrevibacter smithii*, *Bifidobacterium* spp. (including *B. pseudocatenulatum*, *B. animalis* and one unclassified MLG), *Eubacterium* spp. (*E. siraeum*, *E. eligens* and two unclassified *Eubacterium* MLGs) (Tables S2 and S3). Previous studies of a variety of chronic diseases, including type 2 diabetes [19], liver cirrhosis [23], Crohn's disease [24] and ulcerative colitis [25], have also found these bacteria to be enriched in the gut microbiome of healthy control subjects. This suggests that alterations in gut microbiota resulting in decreased relative abundance of these species may be a considerable risk factor for multiple metabolic syndromes, including GDM. Thus, it is conceivable that alterations in the microbiome associated with GDM, contribute to the pathogenesis of GDM.

Other bacteria identified in the current study as being over- or under-represented in GDM patients have also been previously demonstrated to play important roles in the human gut, with potential functional relevance to GDM. For example, patient-enriched *Bacteroides spp.* and *Parabacteroides distasonis* are considered to be opportunistic pathogens in infectious diseases with potential for developing antimicrobial drug resistance [26], and control-enriched *Alistipes* spp. are producers of SCFAs, vitamins and essential amino acids [27]. In addition, striking co-abundance relations were observed between GDM associated MLGs, revealing that the microbes do not function independently. This result suggests that they work cooperatively in maintaining, or interacting with, their environment and in performing various functions that may impact their hosts. Intriguingly, maternal blood glucose levels following an OGTT were positively correlated with the total abundance of patient-enriched MLGs, and negatively correlated with those of control-enriched MLGs, suggesting that the load of some bacteria together may serve a

risk or protective factor for GDM development.

The main limitations of our study are the relatively limited sample size, and the fact that we only analyzed one stool sample per subject, collected in the second trimester of pregnancy. It is well known that immune and metabolic changes occur throughout pregnancy, and that the gut microbiota shifts from first to third trimesters [16]. Consequently, associations between the microbiome and GDM status need to be examined at other time points during pregnancy to provide further insights into when the changes we observed at 21-29 weeks develop, and whether they are sustained for the remainder of the pregnancy. In addition, metadata information available on the effect of maternal GDM status and changes in microbiota composition in pregnancy were limited. Confounding factors such as life style, diet, and antibiotic treatment may further affect both blood glucose levels and gut microbiota composition. In order to more definitively establish the associations observed in the current study, a large cohort investigation, with analysis of other potentially significant variables, will be necessary. Further, the observational, cross-sectional design of the current study precluded examination of potential causality. Whether the microbiome impacts blood glucose levels, glucose levels impact the microbiome, or the relationship is bidirectional, cannot be proven without further experimentation, most likely involving animal models. Furthermore, to identify fecal metagenomic markers with sufficient predictive power to identify GDM, future work will be necessary to refine the diagnostic approach developed in our study, to identify additional markers with improved predictive value and, eventually, to validate them in other larger cohorts.

In summary, our findings extend findings of earlier studies showing a correlation between gut microbiota and various metabolic derangements. Specifically, we demonstrated an important associations between the gut microbiota and GDM. Our results suggest that changes in composition of the gut microbiome may be used to identify individuals with GDM, could potentially be used to identify individuals at risk for GDM, and may contribute to the pathogenesis of GDM.

Methods

Study population and sampling

As part of the Born in Guangzhou Cohort Study (BIGCS), fecal samples were obtained from 124

pregnant women during their second trimester in Guangzhou Women and Children's Medical Center (GWCMC). Pregnant women who had severe obstetric complications (GDM excluded) such as pregnancy-induced hypertension, preeclampsia, or eclampsia, were excluded. Study subjects had not received any antibiotic treatment within 1 month of sample collection and had not ingested yogurt within 2 weeks of sample collection. Samples were frozen immediately (within 30 minutes) and transferred to -80 °C freezers until further analysis.

This study received approval from the Ethics Committee of GWCMC, and written informed consent was obtained from all participating pregnant women. Eligible participants underwent a standard 2h 75g oral glucose tolerance test (OGTT) between 21–29 weeks' gestation by collection of 2ml blood samples fasting, 1h, and 2h after a 75g glucose load, using NaF/EDTA tubes. After centrifugation, plasma glucose was measured by a hexokinase method using Beckman Coulter AU5800 automatic analyzer (Beckman Coulter, California, US). The laboratory previously achieved ISO15189 certification by China National Accreditation Service for Conformity Assessment. GDM was defined using the Chinese diagnostic criteria [28], which is in agreement with the one-step approach endorsed by the American Diabetes Association [29]. Following the OGTT, pregnant women were diagnosed as having GDM if one or more of the following glucose levels were elevated: fasting ≥5.1 mmol/L, 1h ≥10.0 mmol/L, and 2h ≥8.5 mmol/L [28]. None of these women was treated with insulin or glyburide. Maternal age, pre-pregnancy weight and height were extracted from obstetric records of the Hospital Information Systems (HIS) used in GWCMC. Pre-pregnancy body mass index (PBMI) was calculated from height and weight information. Pregnant women were included in this study included 43 patients with GDM (average age 30.5 years; average PBMI 21.9 kg/m^2) and 81 healthy controls (average age 29.1 years; average PBMI 20.2 kg/m^2).

DNA extraction and metagenomic sequencing

Total bacterial DNA was extracted from about 100 mg of feces using Qiagen QIAamp DNA Stool Mini Kit (Qiagen) following the manufacturer's instructions. Extracted DNA of each sample was kept frozen at −20°C until used. Illumina HiSeq 2000 was used to sequence the samples. We constructed a paired-end library with insert size of 350 base pairs (bp) for every sample, and sequenced with 100bp read length from each end. Illumina sequencing reads for fecal samples

from pregnant women were independently processed for quality control using FASTAX Toolkit (http://hannonlab.cshl.edu/fastx_toolkit/). Quality control used the following criteria: (1) reads were removed if they contain more than 3 N bases or more than 50 bases with low quality (<Q20); (2) reads were trimmed in the end with low quality (<Q20) or assigned as N. The remaining reads were then mapped to the human genome using SOAPalinger2 [30] to remove contaminating human DNA. After t QC, an average of 1.9% of low-quality or human genome reads were removed for the 124 samples.

***De novo* assembly, gene calling and gene catalogue construction**

To determine the best assembling method for the obtained high-quality Illumina sequencing reads, we compared the performance of two assemblers, SOAPdenovo v2.04 (as previously used in the MetaHIT and IGC projects) [31, 32] and IDBA-UD v1.1.1 (a *de novo* assembler for metagenomic sequences) [33]. For the SOAPdenovo, we tested the k-mer length ranging from 23bp to 123bp by 10bp step for each sample, and selected the assembled contig set with longest N50 length. For the IDBA-UD, parameters "--mink 21 --maxk 81 --step 20 --pre_correction" were used. For most samples, IDBA-UD obtained a better assembled contig set than SOAPdenovo. This could be attributable to the relative efficiency of IDBA-UD in assembling bacterial genomes within regions of highly uneven depth in metagenomic samples. As a result, we obtained an average 197.9 ± 50.3 Mbp (mean ± SD) contig sets for each pregnant women sample, with N50 length 8.8 ± 3.9 kbp. Unassembled reads from these samples were pooled and re-assembled by using IDBA-UD for further analysis.

Genes were predicted by MetaGeneMark [34] based on parameter exploration by the MOCAT pipeline [35]. A non-redundant gene catalogue of pregnant women samples was constructed using CD-HIT [36], through which, genes with >90% overlap and >95% nucleic acid similarity (no gap allowed) were removed as redundancies. A pregnant women gene catalogue containing 4,344,984 non-redundant genes was generated for fecal samples collected from these 124 pregnant women. This gene catalogue was further combined with the previous integrated gene catalogue (IGC) [32] by removing redundancies in the same manner as above; 2,621,398 genes were removed as redundant. In the end, 39.6% (1,723,586) of the genes in the pregnant women gene catalogue were identified as novel.

Quantification of metagenomic genes

The abundance of genes in the combined non-redundant gene catalogue (combining the pregnant women gene catalogue and IGC) was quantified as relative abundance of reads. First, high-quality reads from each sample were aligned against the gene catalogue using SOAP2.21 [30], with thresholds that allowed a maximum of two mismatches in the initial 32bp seed sequence and 90% similarity over the whole reads. Only two types of alignments were accepted: (1) the entire paired-end read can be mapped onto a gene with the correct insert-size; (2) one end of the paired-end read can be mapped onto the end of a gene, only if the other end of read was mapped outside the genic region. The relative abundance of a gene in a sample was estimated by dividing the number of reads that uniquely mapped to that gene by the length of the gene region and by the total number of reads from the sample that uniquely mapped to any gene in the catalogue. The resulting set of gene relative abundances of a sample was its gene profile.

Richness

We used the gene count and Shannon index to represent the richness and evenness of the gut microbiota for each sample. As defined previously [5], the gene counts of a metagenomic sample were calculated based on their reads mapping number on the non-redundant gene catalogue. To eliminate the influence of sequencing depth fluctuation, an equal number of 11 million reads for all samples were randomly extracted for mapping, and then, the mean number of genes over 30 random drawings was generated. The Shannon index (within sample diversity) was calculated as previously described [19].

Taxonomical and functional analyses

Taxonomical classification of genes. Reference microbial genomes were downloaded from the NCBI-genome database (version May-2015), which included 8,953 bacterial/archaea genomes (of which, 2,785 genomes were complete and 6,168 were draft genomes), and 4,400 viral genomes. Genes from the non-redundant gene catalogue were aligned to reference genomes using BLASTN with parameters “-word_size 16 -evalue 1e-10 -max_target_seqs 5000”. At least 70% alignment coverage of each gene was needed. Based on the parameter exploration of sequence similarity across phylogenetic ranks [37], we used 85% identity as the threshold for genus assignment, and

65% for phylum assignment.

Functional annotation of genes. The Kyoto Encyclopedia of Genes and Genomes (KEGG orthologous, version Apr-2015) and evolutionary genealogy of genes: Non-supervised Orthologous Groups (eggNOG, v4) databases were used for functional annotation of genes. Translated amino acid sequences of genes were searched against these databases using USEARCH v8.0.1616 [38] (evalue < 1e-5, query_cov > 0.70) with a minimum similarity of 30%. Each protein was assigned a KEGG orthologue (KO) or eggNOG orthologue group (OG) based on the best-hit gene in the database. Using this approach, 43.6% and 71.9% of the genes in the combined gene catalogue could be assigned a KO or OG, respectively. As a final step, the abundance profiles of KEGG and eggNOG were calculated by summing up the relative abundance of genes annotated to a feature.

Metagenome-wide association study (MGWAS)

We used the MGWAS methodology to identify gene markers that showed significant abundance differences between the GDM and control individuals. The MGWAS was performed using methodology developed by Qin et al [19]. Briefly, gene relative abundance profiles were initially adjusted for population stratifications using the modified EIGENSTRAT method [39] that allows the use of covariance matrices estimated from abundance levels instead of genotypes. Then, a two-tailed Mann-Whitney U test was performed in the adjusted gene profiles, and the Benjamin-Hochberg procedure [40] was subsequently used to correct the p-values to generate the false discovery rate (FDR, known as “q-value”) for each gene.

Metagenomic linkage group (MLG) analysis

Co-abundance genes were clustered into MLGs based on the previously described methodology [19]. Taxonomic assignment and abundance profiling of the MLGs were performed according to the taxonomy and the relative abundance of their constituent genes as previously described [19]. Briefly, assignment to species requires 90% of genes in an MLG to align with the species’ genome with 95% identity and 70% overlap of query. Assigning an MLG to a genus requires 80% of its genes to align with a genome with 85% identity in both DNA and protein sequences. MLGs were further interconnected according to Spearman’s correlation coefficient ($\rho>0.4$ or $\rho<-0.4$) between

their abundances in all GMD and control samples, and the co-occurrence network of MLGs was visualized by Cytoscape 3.0.2 [41]. The direction of enrichment was determined by the Mann-Whitney U test (p<0.05).

Statistical analysis

Statistical analysis was implemented using the R platform. Distance-based redundancy analysis (dbRDA) was performed using the "vegan" package [42] based on the Bray-Curtis distances on normalized taxa abundance matrices, then visualized using the "ggplot2" package. Permutational multivariate analysis of variance (PERMANOVA) was performed using the "vegan" package, and the permuted *p*-value was obtained by 10,000 permutations.

The Random Forest model has been shown [6] to be a suitable model for exploiting metagenomic data. Random Forest models were trained using the "randomForest" package (default parameters and 10,000 trees) to identify GDM status in a subset of GDM patients and control subjects by using the abundance profiles of species and MLGs. Performance of the predictive model was evaluated with cross-validation error. Variable importance by mean decrease in accuracy was calculated for the Random Forest models using the full set of species or MLGs. By ranking the variables by importance, smaller models were constructed that contained only the most important variables.

Receiver operator characteristic (ROC) analysis was performed using the "pROC" package, we then computed the 95% confidence interval (CI) of the area under the ROC curve (AUC) with 10,000 bootstrap replicates to assess the variability of the measure. Rarefaction analysis was performed to assess the gene richness of metagenomic samples, implemented by in-house Perl scripts.

Reference

1. Ferrara A: **Increasing prevalence of gestational diabetes mellitus: a public health perspective**. *Diabetes care* 2007, **30 Suppl 2**:S141-146.
2. Landon MB, Gabbe SG: **Gestational diabetes mellitus**. *Obstetrics and gynecology* 2011, **118**(6):1379-1393.
3. Kim C, Newton KM, Knopp RH: **Gestational diabetes and the incidence of type 2 diabetes: a systematic review**. *Diabetes care* 2002, **25**(10):1862-1868.
4. Allalou A, Nalla A, Prentice KJ, Liu Y, Zhang M, Dai FF, Ning X, Osborne LR, Cox BJ,

Gunderson EP *et al*: **A Predictive Metabolic Signature for the Transition From Gestational Diabetes Mellitus to Type 2 Diabetes**. *Diabetes* 2016, **65**(9):2529-2539.

5. Le Chatelier E, Nielsen T, Qin J, Prifti E, Hildebrand F, Falony G, Almeida M, Arumugam M, Batto JM, Kennedy S *et al*: **Richness of human gut microbiome correlates with metabolic markers**. *Nature* 2013, **500**(7464):541-546.
6. Karlsson FH, Tremaroli V, Nookaew I, Bergstrom G, Behre CJ, Fagerberg B, Nielsen J, Backhed F: **Gut metagenome in European women with normal, impaired and diabetic glucose control**. *Nature* 2013, **498**(7452):99-103.
7. Karlsson F, Tremaroli V, Nielsen J, Backhed F: **Assessing the human gut microbiota in metabolic diseases**. *Diabetes* 2013, **62**(10):3341-3349.
8. Cani PD, Neyrinck AM, Fava F, Knauf C, Burcelin RG, Tuohy KM, Gibson GR, Delzenne NM: **Selective increases of bifidobacteria in gut microflora improve high-fat-diet-induced diabetes in mice through a mechanism associated with endotoxaemia**. *Diabetologia* 2007, **50**(11):2374-2383.
9. Chu SY, Callaghan WM, Kim SY, Schmid CH, Lau J, England LJ, Dietz PM: **Maternal obesity and risk of gestational diabetes mellitus**. *Diabetes care* 2007, **30**(8):2070-2076.
10. Hedderson MM, Williams MA, Holt VL, Weiss NS, Ferrara A: **Body mass index and weight gain prior to pregnancy and risk of gestational diabetes mellitus**. *American journal of obstetrics and gynecology* 2008, **198**(4):409 e401-407.
11. Pedersen HK, Gudmundsdottir V, Nielsen HB, Hyotylainen T, Nielsen T, Jensen BA, Forslund K, Hildebrand F, Prifti E, Falony G *et al*: **Human gut microbes impact host serum metabolome and insulin sensitivity**. *Nature* 2016, **535**(7612):376-381.
12. Collado MC, Isolauri E, Laitinen K, Salminen S: **Distinct composition of gut microbiota during pregnancy in overweight and normal-weight women**. *The American journal of clinical nutrition* 2008, **88**(4):894-899.
13. Vrieze A, Van Nood E, Holleman F, Salojarvi J, Kootte RS, Bartelsman JF, Dallinga-Thie GM, Ackermans MT, Serlie MJ, Oozeer R *et al*: **Transfer of intestinal microbiota from lean donors increases insulin sensitivity in individuals with metabolic syndrome**. *Gastroenterology* 2012, **143**(4):913-916 e917.
14. Turnbaugh PJ, Ley RE, Mahowald MA, Magrini V, Mardis ER, Gordon JI: **An obesity-associated gut microbiome with increased capacity for energy harvest**. *Nature* 2006, **444**(7122):1027-1031.
15. England LJ, Dietz PM, Njoroge T, Callaghan WM, Bruce C, Buus RM, Williamson DF: **Preventing type 2 diabetes: public health implications for women with a history of gestational diabetes mellitus**. *American journal of obstetrics and gynecology* 2009, **200**(4):365 e361-368.
16. Koren O, Goodrich JK, Cullender TC, Spor A, Laitinen K, Backhed HK, Gonzalez A, Werner JJ, Angenent LT, Knight R *et al*: **Host remodeling of the gut microbiome and metabolic changes during pregnancy**. *Cell* 2012, **150**(3):470-480.
17. Gomez-Arango LF, Barrett HL, McIntyre HD, Callaway LK, Morrison M, Dekker Nitert M, Group ST: **Connections Between the Gut Microbiome and Metabolic Hormones in Early Pregnancy in Overweight and Obese Women**. *Diabetes* 2016, **65**(8):2214-2223.
18. Wang J, Jia H: **Metagenome-wide association studies: fine-mining the microbiome**. *Nature reviews Microbiology* 2016, **14**(8):508-522.

19. Qin J, Li Y, Cai Z, Li S, Zhu J, Zhang F, Liang S, Zhang W, Guan Y, Shen D *et al*: **A metagenome-wide association study of gut microbiota in type 2 diabetes**. *Nature* 2012, **490**(7418):55-60.
20. Lowe WL, Jr., Scholtens DM, Sandler V, Hayes MG: **Genetics of Gestational Diabetes Mellitus and Maternal Metabolism**. *Current diabetes reports* 2016, **16**(2):15.
21. Garrett WS, Gallini CA, Yatsunenko T, Michaud M, DuBois A, Delaney ML, Punit S, Karlsson M, Bry L, Glickman JN *et al*: **Enterobacteriaceae act in concert with the gut microbiota to induce spontaneous and maternally transmitted colitis**. *Cell host & microbe* 2010, **8**(3):292-300.
22. Liu J, Yan Q, Luo F, Shang D, Wu D, Zhang H, Shang X, Kang X, Abdo M, Liu B *et al*: **Acute cholecystitis associated with infection of Enterobacteriaceae from gut microbiota**. *Clinical microbiology and infection : the official publication of the European Society of Clinical Microbiology and Infectious Diseases* 2015, **21**(9):851 e851-859.
23. Qin N, Yang F, Li A, Prifti E, Chen Y, Shao L, Guo J, Le Chatelier E, Yao J, Wu L *et al*: **Alterations of the human gut microbiome in liver cirrhosis**. *Nature* 2014, **513**(7516):59-64.
24. Gevers D, Kugathasan S, Denson LA, Vazquez-Baeza Y, Van Treuren W, Ren B, Schwager E, Knights D, Song SJ, Yassour M *et al*: **The treatment-naive microbiome in new-onset Crohn's disease**. *Cell host & microbe* 2014, **15**(3):382-392.
25. Machiels K, Joossens M, Sabino J, De Preter V, Arijs I, Eeckhaut V, Ballet V, Claes K, Van Immerseel F, Verbeke K *et al*: **A decrease of the butyrate-producing species Roseburia hominis and Faecalibacterium prausnitzii defines dysbiosis in patients with ulcerative colitis**. *Gut* 2014, **63**(8):1275-1283.
26. Boente RF, Ferreira LQ, Falcao LS, Miranda KR, Guimaraes PL, Santos-Filho J, Vieira JM, Barroso DE, Emond JP, Ferreira EO *et al*: **Detection of resistance genes and susceptibility patterns in Bacteroides and Parabacteroides strains**. *Anaerobe* 2010, **16**(3):190-194.
27. Shetty SA, Marathe NP, Lanjekar V, Ranade D, Shouche YS: **Comparative genome analysis of Megasphaera sp. reveals niche specialization and its potential role in the human gut**. *PloS one* 2013, **8**(11):e79353.
28. Zhu WW, Yang HX: **Diagnosis of gestational diabetes mellitus in China**. *Diabetes Care* 2013, **36**(6):e76.
29. Association. AD: **Standards of medical care in diabetes--2011**. *Diabetes Care* 2011, **34 Suppl 1**:S11-61.
30. Li R, Yu C, Li Y, Lam TW, Yiu SM, Kristiansen K, Wang J: **SOAP2: an improved ultrafast tool for short read alignment**. *Bioinformatics* 2009, **25**(15):1966-1967.
31. Li R, Zhu H, Ruan J, Qian W, Fang X, Shi Z, Li Y, Li S, Shan G, Kristiansen K *et al*: **De novo assembly of human genomes with massively parallel short read sequencing**. *Genome Res* 2010, **20**(2):265-272.
32. Li J, Jia H, Cai X, Zhong H, Feng Q, Sunagawa S, Arumugam M, Kultima JR, Prifti E, Nielsen T *et al*: **An integrated catalog of reference genes in the human gut microbiome**. *Nature biotechnology* 2014, **32**(8):834-841.
33. Peng Y, Leung HC, Yiu SM, Chin FY: **IDBA-UD: a de novo assembler for single-cell and metagenomic sequencing data with highly uneven depth**. *Bioinformatics* 2012, **28**(11):1420-1428.
34. Zhu W, Lomsadze A, Borodovsky M: **Ab initio gene identification in metagenomic**

sequences. *Nucleic acids research* 2010, **38**(12):e132.

35. Kultima JR, Sunagawa S, Li J, Chen W, Chen H, Mende DR, Arumugam M, Pan Q, Liu B, Qin J *et al*: **MOCAT: a metagenomics assembly and gene prediction toolkit**. *PloS one* 2012, **7**(10):e47656.
36. Li W, Godzik A: **Cd-hit: a fast program for clustering and comparing large sets of protein or nucleotide sequences**. *Bioinformatics* 2006, **22**(13):1658-1659.
37. Arumugam M, Raes J, Pelletier E, Le Paslier D, Yamada T, Mende DR, Fernandes GR, Tap J, Bruls T, Batto JM *et al*: **Enterotypes of the human gut microbiome**. *Nature* 2011, **473**(7346):174-180.
38. Edgar RC: **Search and clustering orders of magnitude faster than BLAST**. *Bioinformatics* 2010, **26**(19):2460-2461.
39. Price AL, Patterson NJ, Plenge RM, Weinblatt ME, Shadick NA, Reich D: **Principal components analysis corrects for stratification in genome-wide association studies**. *Nature genetics* 2006, **38**(8):904-909.
40. Storey JD: **A direct approach to false discovery rates Journal of the Royal Statistical Society: Series B (Statistical Methodology) Volume 64, Issue 3**. *Journal of the Royal Statistical Society: Series B (Statistical Methodology)* 2002, **64**(3):479-498.
41. Shannon P, Markiel A, Ozier O, Baliga NS, Wang JT, Ramage D, Amin N, Schwikowski B, Ideker T: **Cytoscape: a software environment for integrated models of biomolecular interaction networks**. *Genome research* 2003, **13**(11):2498-2504.
42. Dixon P: **VEGAN, a package of R functions for community ecology Journal of Vegetation Science Volume 14, Issue 6**. *Journal of Vegetation Science* 2003, **14**(6):927-930.

Acknowledgements

We thank all the pregnant women who participated in the Born in Guangzhou Cohort Study and all staff in the cohort team for their contribution to this study, particularly the research nurses and midwives and other recruiting staff for their excellent work.

This study is supported by the National Natural Science Foundation of China (81673181) and Guangzhou Science and Technology Bureau, Guangzhou, China (201508030037).The sponsors had no role in design and conduct of the study; collection, management, analysis, and interpretation of the data; or in preparation, review, or approval of the manuscript; or the decision to submit the manuscript for publication.

Author contributions

XQ and HX designed the birth cohort on which this study was based. XQ and HX designed the study and directed its implementation. YG, YK, MY, JH, JL, NC, WX, SS, LQ, YW, CH, QC, WL and YW were involved in study design and sample collection. YG, YK and SL analyzed the data and drafted the manuscript. XQ, HD, JL and CP revised the manuscript. All authors critically revised the manuscript, and approved the final version.

Figure legends

Figure 1 | Difference in microbial composition between GDM and healthy pregnant women. (**a**) Distance-based redundancy analysis (dbRDA) based on Bray–Curtis distances between microbial genera, revealing a GDM dysbiosis which overlaps only in part with taxonomic composition in GDM patients and healthy controls. The first two principal components (PCs) and the ratio of variance contributed by them is shown. Lines connect samples in the same group, and coloured circles cover the samples near the center of gravity for each group. (**b**) Boxplot shows genera that differ significantly between GDM patients and healthy controls. Genera with $q<0.05$ (Mann-Whitney U test corrected by the Benjamini-Hochberg method) are shown. Red and green boxes represent GDM patients and healthy controls, respectively. Only the genera with average relative abundances greater than 0.05% of total abundance in all samples are shown for clarity. The boxes represent the interquartile range (IQR) between first and third quartiles and the line inside represents the median. The whiskers denote the lowest and highest values within 1.5 times

IQR from the first and third quartiles, respectively. The circles represent outliers beyond the whiskers.

Figure 2 | Interconnection of GDM-associated MLGs. A co-occurrence network deduced from GDM-enriched (red circle) and control-enriched (green circle) MLGs is shown. Nodes depict MLGs with their ID displayed in the center. The size of each node indicates the number of genes within the MLG. Connecting lines represent Spearman correlation coefficient ρ >0.40 (represented by gray line) or <-0.40 (represented by red line). Only MLGs with >200 genes or >0.1% relative abundance are shown for clarity of presentation and visualization. GDM-enriched MLGs for the Enterobacteriaceae group is labeled.

Figure 3 | Association of gross abundance of GDM-enriched and control-enriched MLGs with blood glucose levels 0, 60, and 120 minutes after an oral glucose tolerance test. Scatter plots of samples are shown with lines indicating linear fit.

Figure 4 | Correlation of blood glucose levels 0, 60, and 120 minutes after an oral glucose tolerance test, and HbA1c levels with MLGs (a) and species (b). Spearman's rank correlation coefficients and P-values for the correlations are shown. '+' denotes P<0.05; '++' denotes P<0.01. Only MLGs or species with average relative abundances greater than 0.001% and correlated (P<0.05) with at least one index are shown for clarity.

Figure 5 | Association of microbial genetic functional pathway composition in GDM patients and healthy pregnant women. (a) Distributions of relative abundances of KEGG pathway categories in GDM patients and healthy controls. '*' denotes q<0.05 (Mann-Whitney U test corrected by the Benjamini-Hochberg method) (b) Correlation of blood glucose levels 0, 60, and 120 minutes after an oral glucose tolerance test, and HbA1c levels, with PTS system and LPS biosynthesis and transport system. Spearman's rank correlation coefficients and P-values for the correlations are shown. '+' denotes P<0.05; '++' denotes P<0.01.

Figure 6 | Classification of GDM status by the relative abundance of MLGs and species. (a)

Classification performance of a random forest model using MLG or species abundance assessed by AUC. The performance was explored for different numbers of explanatory variables, ordered in importance. (b-c) The 30 most discriminant MLGs (b) and species (c) in the models classifying GDM and controls. The bar lengths in b and c indicate the importance of the variable, and colors represent enrichment in GDM (red shades) or controls (blue shades). (d) ROC analysis for classification of GDM status by MLGs and PBMI.

Additional material

Additional file1:

File format: .xlsx

Table S1: **Phenotype information of 43 GDM and 81 healthy pregnant women**

Table S2: **Bacterial species that differed significantly between two cohorts.**

Table S3: **Detailed information of 129 GDM-associated MLGs**

Additional file 2:

File format: .docx

Figure S1: **Density histogram showing the P-value distribution between GDM patients and healthy pregnant women for all genes tested.** The horizontal line represents the expected distribution of P-values, and the π0 value indicates the proportion of genes under the null hypothesis.

Figure S2: **Figure S2 | Correlation between Enterobacteriaceae relative abundance and PBMI.** Scatter plots of samples are shown with lines indicating linear fit.

Figure S3: **Figure S3 | Classification of GDM status by abundance of MLGs and PBMI.** The 30 most discriminant MLGs or PBMI in the models for classifying GDM and controls. The bar lengths indicate the importance of the variable, and colors represent enrichment in GDM (red shades) or controls (blue shades)

Figure 1

Click here to download Figure Figure 1.pdf

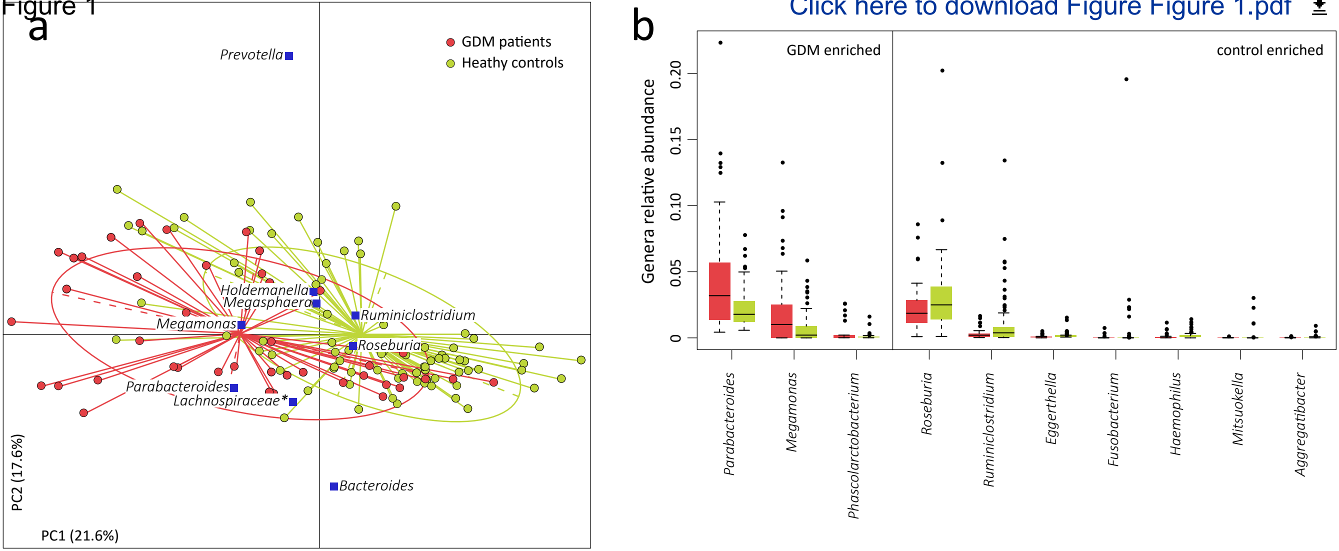

Figure 2

Click here to download Figure Figure 2.pdf

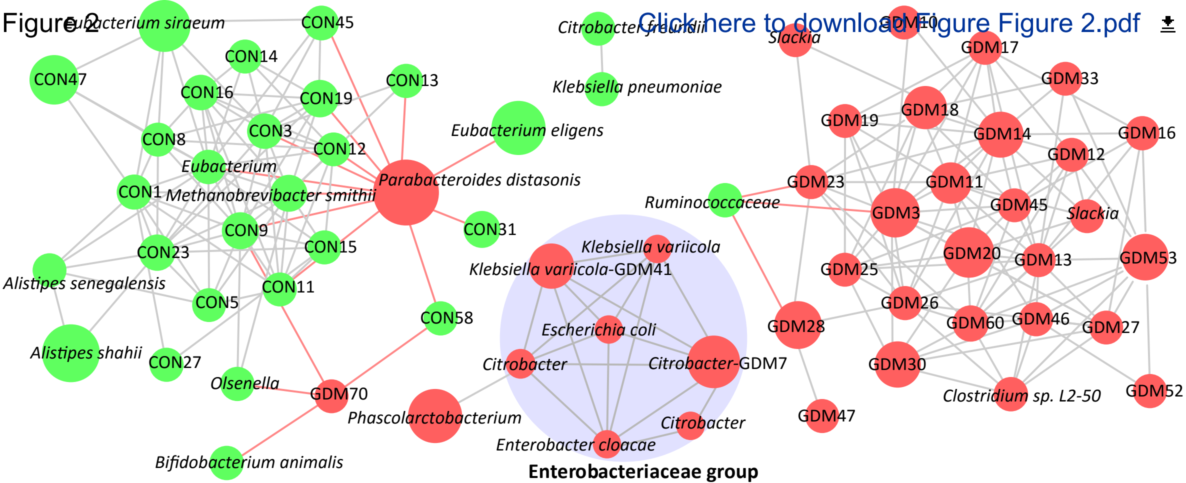

Figure 3

Click here to download Figure Figure 3.pdf

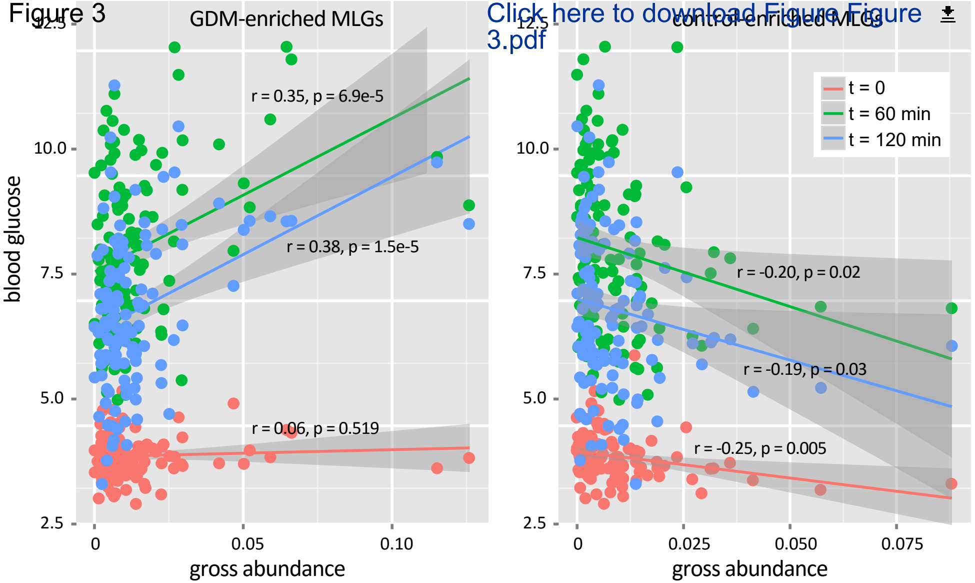

Figure 4

Click here to download Figure Figure 4.pdf

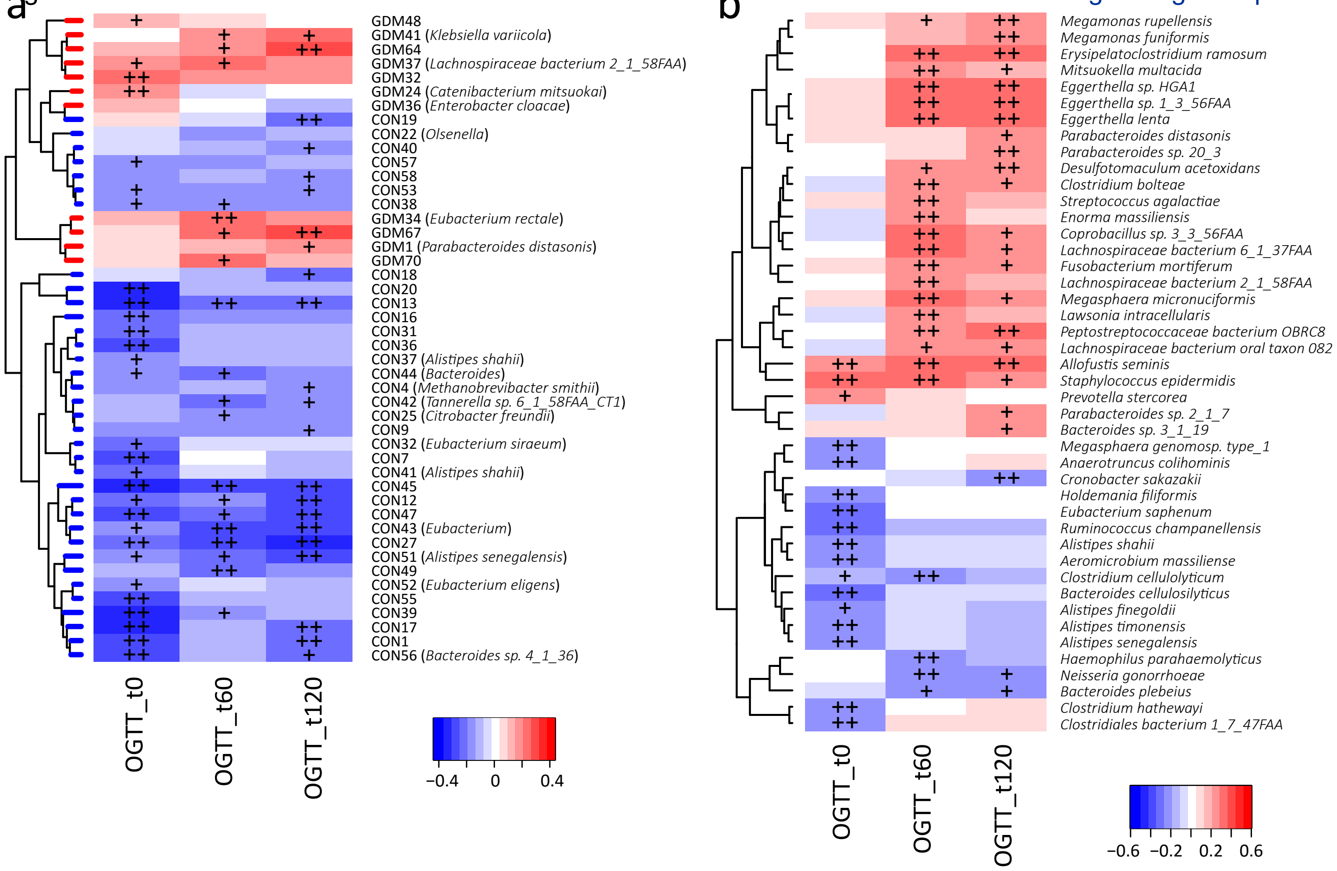

Figure 5

Click here to download Figure Figure 5.pdf

a

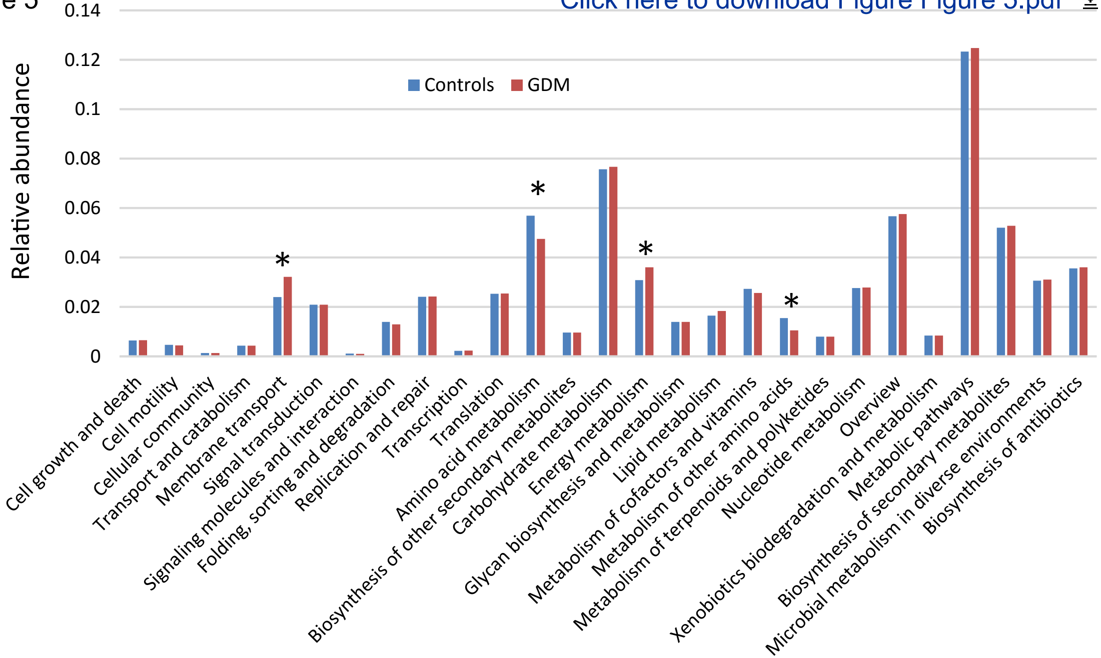

b

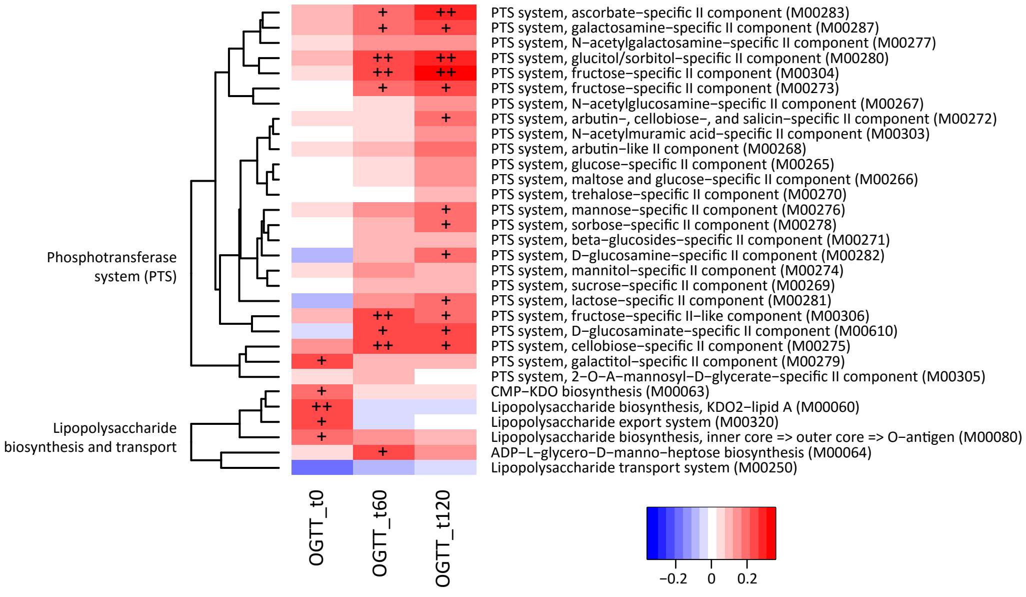

Figure 6

Click here to download Figure Figure 6.pdf

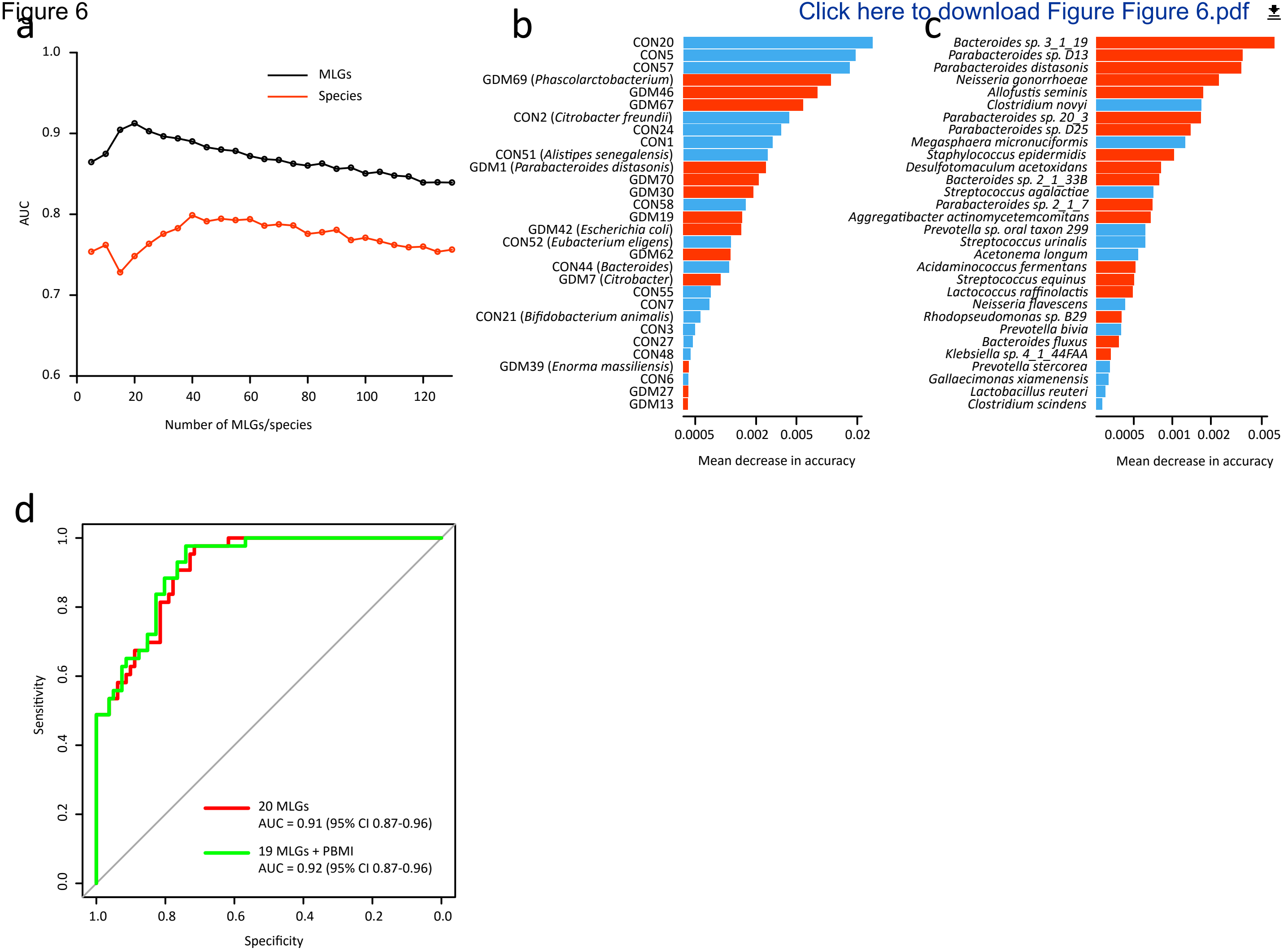

Additional file 1

Click here to access/download
Supplementary Material
Additional file 1.xlsx

Additional file2

Click here to access/download
Supplementary Material
Additional file2.docx

cover letter Click here to download Personal Cover cover letter-1.docx

Dear Dr. Laurie Goodman,

Enclosed please find our manuscript entitled "**Connections between human gut microbiome and gestational diabetes mellitus**" for your consideration of publication as an Article in ***GigaScience***.

Emerging evidence has revealed a link between the gut microbiome and human metabolic health. The gut microbiome can modulate metabolic health and affect insulin resistance, and may play an important role in the etiology of gestational diabetes mellitus (GDM). However, studies focusing on changes in the gut microbiome during pregnancy and development of GDM have not been reported so far.

In the present study, we performed whole-metagenome shotgun sequencing analyses of the gut microbiome during pregnancy to explore associations between GDM and the composition and abundance of microbial taxonomic units and functional genes (43 GDM patients versus 81 healthy control subjects). The objective was to obtain a comprehensive understanding of the gut microbiome's role in the etiopathogenesis of GDM.

Our data showed that GDM patients had significantly higher abundance of *Parabacteroides*, *Megamonas* and *Phascolarctobacterium*. Metagenome-wide association study (MGWAS) identified 154,837 genes with significant abundance differences between GDM patients and healthy pregnant women. Furthermore, the GDM microbiome showed greater abundance of membrane transport and energy metabolism pathways, involving the phosphotransferase system, and the lipopolysaccharide biosynthesis and export system, that may contribute to the potential relationship between gut microbes and blood glucose tolerance. Our data also imply that fecal metagenome linkage groups may provide a mechanism and biomarkers for early detection of GDM or, potentially, for identifying risk of developing GDM.

We believe that findings from our study represent an novel relationship between gut microbiome composition and GDM status, by which the gut microbiome may potentially be used to identify individuals at risk for GDM, and may contribute to the pathogenesis of GDM. And as such should

be of great interest to the general medical readership of your journal.

All authors have reviewed and approved the final version. Neither this manuscript, nor one with similar content, has been published or is being considered for publication elsewhere. None of the authors have conflict of interest with regard to the data presented in this manuscript.

Thank you for your time and consideration. We look forward to further discussion regarding this manuscript.

Respectfully Yours,

Xiu Qiu, M. D., Ph. D.
Division of Birth Cohort Study,
Guangzhou Women and Children's Medical Center, Guangzhou Medical University
9 Jinsui Road, Tianhe District, Guangzhou 510623, China
Tel: 86 2038367162
Fax: 86 2038367162
Email: qxiu0161@163.com
