# Supplementary material for: Connections between the human gut microbiome and gestational diabetes mellitus
Source: Gigascience. 2017 Jul 31;6(8):1–12. doi: 10.1093/gigascience/gix058 (PMC5597849; doi:10.1093/gigascience/gix058)
Supplement: Additional Files [file gix058_Supp.zip › Additional file2.docx]

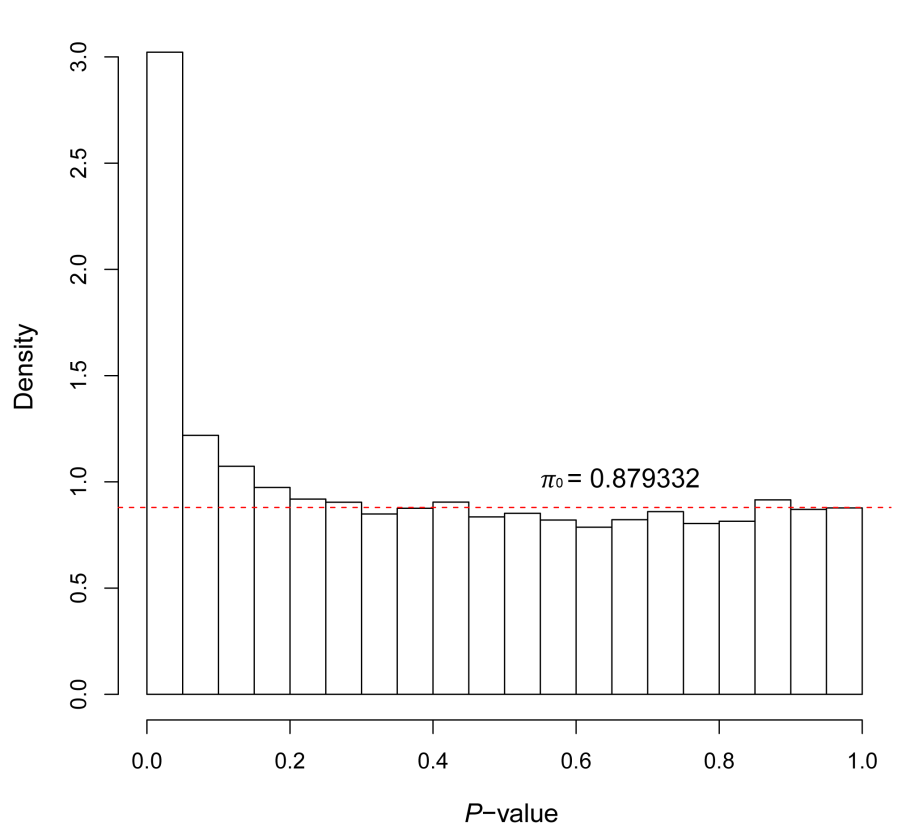


**Figure S1 | Density histogram showing the P-value distribution between GDM patients and healthy pregnant women for all genes tested.** The horizontal line represents the expected distribution of P-values, and the π0 value indicates the proportion of genes under the null hypothesis.


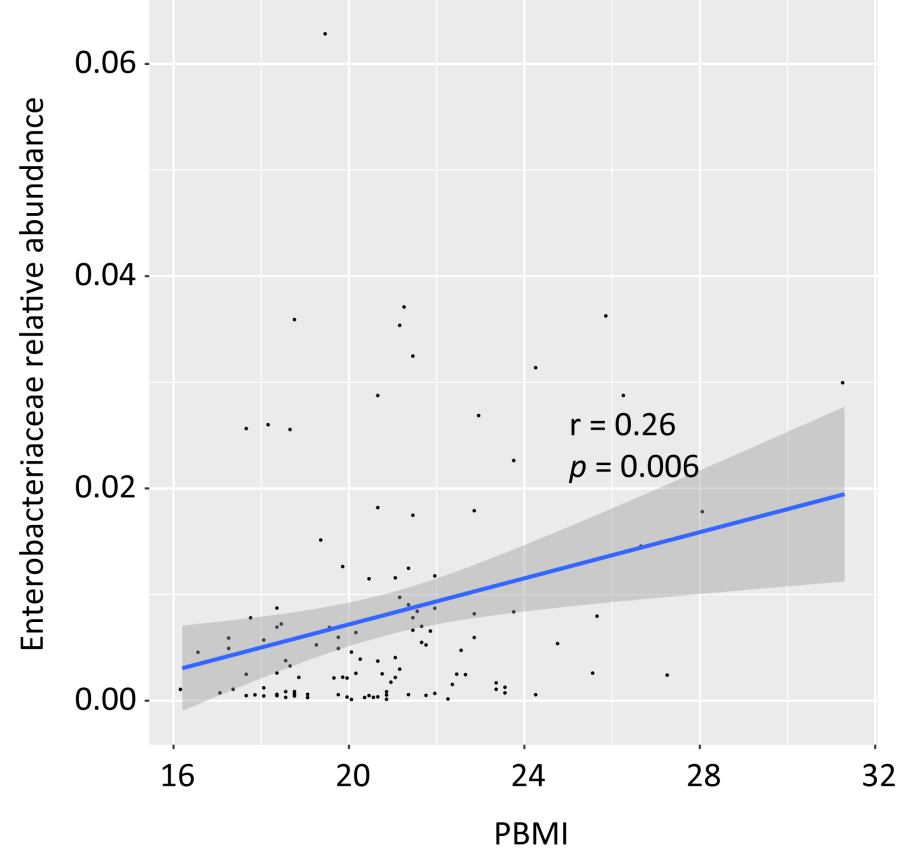


**Figure S2 | Correlation between Enterobacteriaceae relative abundance and PBMI.** Scatter plots of samples are shown with lines indicating linear fit.


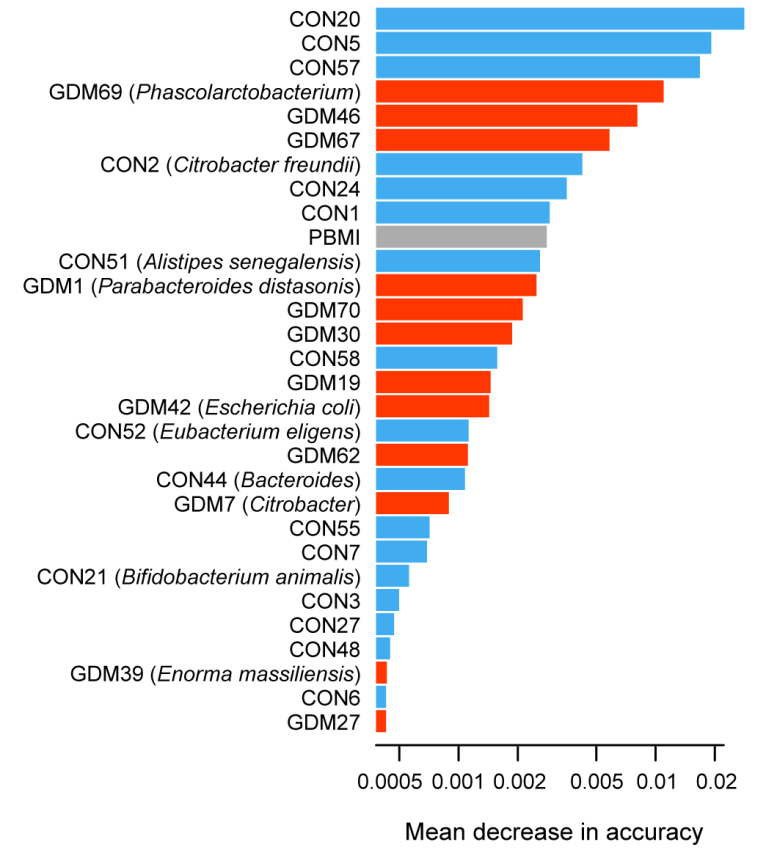


**Figure S3 | Classification of GDM status by abundance of MLGs and PBMI.** The 30 most discriminant MLGs or PBMI in the models for classifying GDM and controls. The bar lengths indicate the importance of the variable, and colors represent enrichment in GDM (red shades) or controls (blue shades).
