# Supplementary material for: Connections between the human gut microbiome and gestational diabetes mellitus
Source: Gigascience. 2017 Jul 31;6(8):1–12. doi: 10.1093/gigascience/gix058 (PMC5597849; doi:10.1093/gigascience/gix058)
Supplement: Response-to-Reviewer-Comments_Original-Submission.pdf [file gix058_Response-to-Reviewer-Comments_Original-Submission.pdf]

Author's Response To Reviewer Comments

Replies to comments of Reviewers

Reviewer #1

This article describes the first metagenomic analysis of the gut microbiome in women with and without GDM. It is well-conducted, has generated fascinating data and will be of interest to people within the microbiome field and also in the diabetes field.

Major comments
* Methods-participant description: The description of the participant cohort is missing some important information: Please add to the table: birth weight of the infants, gestational age at delivery and mode of delivery.
# Reply: Thank you for your comments. We have added the description on birth weight of the infants, gestational age at delivery and mode of delivery in supplemental table S1.

* In addition, please provide more information on whether other disease states some of which may affect either glucose metabolism or microbiome composition such as thyroid disorders, asthma especially when treated with glucocorticoids, inflammatory bowel disease were excluded from the study as well.
# Reply: Thanks for your comments. In this study, we did not include women with pre-pregnancy diabetes, hypertensive disorders, thyroid disorders and asthma. We have presented more details about the study design, criteria of inclusion in Method section. Please refer to page 9 line 19-30:
“As part of the Born in Guangzhou Cohort Study (BIGCS) [1], fecal samples were obtained from 298 pregnant women during their second trimester in Guangzhou Women and Children’s Medical Center (GWCMC) between 1st August, 2012 and 31st Aug, 2013. The inclusion criteria of current study was as follows: 1) without diseases which might affect glucose metabolism or microbiome composition such as pre-pregnancy diabetes, hypertension, thyroid disorders, asthma, lipid metabolic disorders, inflammatory bowel disease, irritable bowel syndrome and celiac disease; 2) had not received any antibiotic treatment 1 month before sample collection; 3) had not taken probiotics 2 weeks before sample collection. Of the 287 eligible women, 43 had a diagnosis of GDM and were included in the present study as the case group, and 81 women of non-GDM were randomly selected as the control group. Basic characteristics of the 124 pregnant women included in the study are summarized in Table S1. Fecal samples were frozen at -20°C freezers immediately (within 30 minutes) and transferred to -80 °C freezers within 24 hours after collected.”

* There was a wide range in the gestational age at which fecal samples were collected and also when OGTTs were performed. Furthermore, the fecal samples were not consistently collected at the same timepoint as the OGTT. It is possible that women who had an OGTT at 21 weeks gestation were normoglycemic at that time but crossed the threshold later and would have had GDM if they had an OGTT at 28 weeks. Has this been checked and what was the reason for the wide range in timing of OGTT testing? For women who had a large difference between OGTT and decal sample, for instance C008 and C112, this may be especially important.

# Reply: The ideal method for fecal samples collection would be on-site collection when the OGTT testing is performed. However, big challenges exist for this strategy in clinics. In our study, fecal sample was acceptable in following either ways: (1) collected on-site when OGTT was performed, or (2) collected at home before acquisition of the OGTT results, frozen at -20°C freezers immediately (within 30 minutes) and transferred to -80 °C freezers in our hospital within 24 hours after collected. Thus, there might be a substantial time lag between fecal sample collection and OGTT testing in some cases as noted. Furthermore, during this lag, the participants did not know the results of OGTT, so they would not change their diet or incur any other intervention.
The OGTT test is usually carried out between 24 weeks and 28 weeks' gestation. Because of the timing of clinical appointment, some participants might be offered the test earlier or later, which resulted in a wide range in timing of OGTT test in our study (21-29 weeks).
The GDM diagnosis was made according to the Chinese clinical standards using OGTT results and confirmed using diagnosis information from medical records after delivery.

* Also for C189, no OGTT was performed, how was ascertained that she was C and not GDM?
# Reply: Thank you for your comment. We rechecked the medical record and located the OGTT result (t0= 4.26, t60=7.67, t120=6.54) for the participant C189.

* Methods—taxonomical classification of genes. The cut-off for genus identity at 85% and is much lower than what is commonly used: 95-97% of genus and is lower than what is usually considered the threshold for family and even order. Please explain why this threshold was used.
# Reply: The nucleic acid identity cut-off 95-97% is often used to genus level taxonomy assignment in studies based on 16S rDNA sequencing. In our metagenomics study, we followed the previous studies, performed the parameter exploration for taxonomy assignment of the common microbial genes and identified the best nucleic acid similarity of 85% and 65% for genus and phylum level classification (see Figure S1 in Arumugam M. et al. 2011 and supplemental information in Qin J. et al.2012)[2] [3].

* Methods—statistical analysis. Since in house Perl scripts were used for the rarefraction analysis, have these scripts been validated against other scripts to ensure that they are valid?
# Reply: Yes, these scripts have been validated and used for analysis in earlier published metagenomic studies [3, 4].

* In Figure 1A, it is not clear what the bacteria are that are denoted in blue. These are interesting bacteria which have been associated with different functions. Does the denotation in blue mean that these are not associated with either GDM or control? If so, why are they included?
# Reply: The missing information has been incorporated into the Figure legend. The blue square mean that genera as the main contributors are plotted by their loadings in these principal components.

* In figure 2, the representation of the co-occurrence of MLGs in the two groups is not optimal. It is understandable that the bacterial names are not displayed in the figure but since they are only available in the supplementary material, figure 2 becomes almost meaningless since except for a few bacteria, the other connections are black boxes (or perhaps more appropriately red and green circles). I would like to suggest to the authors to perhaps include as part of figure 2 a list of

the bacteria representing the clusters either in the legend or in the figure itself. That would make the figure more informative as a stand-alone figure and would obviate the need to find the information in the supplementary material.
# Reply: Thank you for your kind comment. We have put the bacteria representing the clusters in the new Figure 2.

* In figure 3, were the GDM-enriched MLGs also correlated with the glucose measures of the control individuals and vice versa? This would be interesting data since it would give an insight into whether the relationship of the bacteria to the glucose levels is independent of the disease state or part of the physiological process.
# Reply: Thank you for your suggestion and sorry for confusion. After careful consideration, we decided to modify the presentation of Figure 3. In our study, 68% of the significant different genes were clustered into 129 metagenomic linkage groups (MLGs). 71 of them were enriched in GDM patients while the rest were enriched in control group. In this revision, we used the ratio of GDM-enriched and controls enriched MLGs to illustrate the microbiome dysbiosis. The revised Figure 3 demonstrated the ratio of GDM-enriched and control-enriched MLGs were positively correlated with the blood glucose tolerance levels in total population (n=124), which is consistent with the results of original Figure 3.

* It is possible that there is a difference in gut microbiome composition in those women with GDM that were diagnosed based on mainly on their Fasting glucose levels vs only their 1 or 2 hour levels. Has this been checked?
# Reply: Yes, we did check and find that there are no difference in gut microbial composition in GDM subjects who had impaired fasting glucose (abnormal fasting plasma glucose) and isolated impaired glucose tolerance (normal fasting plasma glucose) (p=0.581).

* It was my impression from figure 1b that Aggregibacter was enriched in the control women, however in figure 6c it is shown as enriched in GDM. Please check this and of course also for the other bacteria mentioned.
# Reply: Thank you for identifying this inaccuracy. At genus level, we found that the genus Aggregibacter was significantly enriched in control subjects while at the species level, one species A. actinomycetemcomitans was enriched in GDM patients (due to the average relative abundance is very low, this species was not shown at Table S2).

* It could be argued too that the model to predict GDM should be compared with a prediction model that is based on easy to measure clinical parameters including prepregnancy BMI, family history of diabetes, a glucose measure (either glucose or HbA1c), gestational weight gain, rather than just comparing it to a microbiome explanatory model. The explanatory model is very effective (explaining >90% of variation) but for a fair comparison, especially if gut microbiome composition would be used for prediction, would be against commonly used clinical parameters. Furthermore, since the samples were taken at the time of OGTT, taking a blood sample and measuring blood glucose could be argued is easier and cheaper. Also since this is a cross-sectional microbiome analysis, it is not clear from these results whether or not the women

developing GDM developed the gut dysbiosis in pregnancy or whether it was present pre-pregnancy. I think that therefore the first and concluding paragraph of the discussion should be reworded to include this. This does not distract from the value of the study since it points to bacteria which may be implicated in the pathogenic process of GDM.
Reply: Thanks for your comment. We agree with your point that using clinical parameters and blood glucose for GDM prediction would be easier and cheaper at the moment. The main goal of current study was to examine the connection between microbiome and GDM, rather than predicting GDM using microbiome. The predictive ability of microbiome, especially in comparison or in combination with other easily assessed clinical measurements, will be evaluated in more detail in the future study. We also agree that our study was cross-sectional and unable to determine the causal relationship between microbiome and GDM.
Since this is a cross-sectional microbiome analysis, there are several limitations in our study. We add some discussion on this issue, please refer to page 9, line 1-11:
“There were several limitations in our study. First, the sample size is relatively small. Second, we only analyzed one stool sample per participant, which was collected in the second trimester of pregnancy. It is well known that immune and metabolic changes occur throughout pregnancy, and that the gut microbiota shifts from first to third trimesters [5]. In the present study, we are unable to clarify the causal relationship between the microbiome and the development of GDM due to the cross-sectional design. Consequently, data at multiple time points are needed to provide further insights into their dynamic relationship. Third, we did not have information on several factors such as life style and diet may further affect both blood glucose levels and gut microbiota composition. In order to more confirm the associations observed in the current study, a large prospective cohort investigation, with analysis of other potentially significant variables, will be necessary. ”

Reviewer #2: The authors have done a good work studying the differences between healthy and gestational diabetes mellitus pregnant women. It is an interesting approach due to there is scarce data showing the connections between gut microbiota and GDM. However, the authors have not been able to build a story with the good results they have.
Although the authors stated "In this study, we used whole-metagenome shotgun sequencing analyses of the gut microbiome during pregnancy to explore associations between GDM and the composition and abundance of microbial taxonomic units and functional genes. The objective was to obtain a comprehensive understanding of the gut microbiome's role in the etiopathogenesis of GDM", the opinion of this reviewer is that this aim has not been accomplished. The authors lack in relating all the data together, showing only that these groups of bacteria increase or decrease, resulting in a classical study similar to the 16S done until the date. Thus, although the authors, using elegant bioinformatics approaches, have shown new and relevant data for the elucidation of the gestational diabetes mellitus, the result is a manuscript without the relevancy to be published in Gigascience, at least in this current form.
Reply: Thank you very much for your comments. In our original version of manuscript, we identified abundance of bacteria was different between GDM and the controls. Following your suggestions, we have proposed a metabolic pathway based on our findings and discussed it in-depth in the Discussion section.

Some suggestions and comments are listed below:
A general feeling on the whole manuscript is that the authors seem that always leave work to be

done in the future. There are many sentences like this "This intriguing observation warrants further studies". These sentences are in part responsible of my opinion of the manuscript, due to many of these comments are easy to measure, or the authors already have the information.

* When human samples are analyzed, it is important to show the characteristics of the study subjects. Humans are not homogeneous experimental animals. Many variables may obstruct the actual result.
# Reply: Thank you for your comment. Basic characteristics of the 124 pregnant women included in the study are summarized in new Table S1.

* The authors showed in the results section that LPS and PTS systems were associated to the glucose tolerance levels, but in the discussion section, the authors do not explain anything about this relationship.
# Reply: Thank you for your comment. We have re-organized the discussion section and add some discussion about the potential mechanism of LPS and PTS systems, please refer to page 8 line 12-30:
"Functional analysis showed that the LPS biosynthesis and export systems were involved in regulation of glucose levels. Previous studies have shown that the higher systemic LPS levels were associated with low-grade chronic inflammation in obesity, metabolic syndrome and type 2 diabetes [6-8]. Based on current knowledge, the possible pathways linking LPS levels to glucose metabolism may include the increases in intestinal permeability, the changes in the relative amounts of gram negative vs. gram positive bacteria and a low-grade chronic inflammatory state. LPS is a bacterial cell wall component in gram-negative bacteria and can stimulate an inflammatory response [9, 10]. Gut microbiome dysbiosis can facilitate LPS entry into the systemic circulation through increasing gut permeability, which leads to inflammation and metabolic dysfunction [11]. Our results were concordant with a previous report [3] which found that gut microbiota dysbiosis in type 2 diabetes was characterized by a decrease in gram-positive butyrate producing Clostridium species that lack LPS and an increase in gram-negative opportunistic pathogens including some Bacteroidetes and Proteobacteria species that contain LPS. The functional analysis in the present study found that membrane transport, energy metabolic and PTS pathways were enriched in the GDM patients. PTS pathways are responsible for transporting glucose through outer and inner membranes and catalyzing the uptake of carbohydrates. The increased relative abundance of these pathways may indicate gut environment of a GDM status may stimulate bacterial accelerated usage of glucose as energy."

Particular comments:
* Page5, line 2: What about the family level?
# Reply: Thank you for your reminding. We missed the descriptions of microbial composition difference at the family level. We have rewritten the sentence "however, the order Clostridiales and the families Enterobacteriaceae, Coriobacteriaceae were enriched in healthy controls." (Page 5, line 6-8)

* Page5, line 34: What are MLGs? you have not indicated it
# Reply: Metagemomic linkage group (MLG) is defined as a group of genetic materials in a metagenome that is probably physically linked as a unit rather than being independently distributed in their relative abundances. We have added information to clarify this point, and

made a brief description in the methods. (Page 5, line 22-23)

* Page5, line 58: pro or pre-gestational body mass?
# Reply: We have corrected the word as pre-pregnancy body mass index. Please refer to Page 6, line 5.

* Page6, line 44: It would be interesting to measure LPS amount if you have plasma samples. In this way, the authors could establish this assumption
# Reply: Thank you for your insightful comment. Unfortunately, we did not store blood sample when OGTT was performed so that we could not perform the LPS tests.

* Page6, line 48: This sentence (the last one) is part of the discussion section.
# Reply: Thank you. In light of your comments, we have re-organized the discussion section.

* Page7, line23: This is part of the conclusions
# Reply: Thank you. In light of your comments, we have re-organized the discussion section..

* Page7, lines 44-54: This information has already mentioned in the results section
# Reply: Thank you. We have deleted it to avoid redundancy.

* Page8, line8: Which are the metabolic roles of these bacteria? the authors have the information
# Reply: In this revision, we provided information this point in the discussion section (Page 7,line 27-Page 8,line 9).
"The family Enterobacteriaceae also occurred with a higher relative abundance in GDM patients than in healthy controls, which indicates a status of gut flora dysbiosis that may lead to a series of chronic diseases, such as colitis [12], Crohn's disease and acute cholecystitis [13]. Previous studies have shown that Enterobacteriaceae instigate inflammation to induce colitis [12], and the endotoxin–producing bacterium Enterobacter contributed to the development of obesity in gnotobiotic mice [14].
The decreased microbes in GDM patients included Bifidobacterium spp. (including B. pseudocatenulatum, B. animalis and one unclassified MLG), Eubacterium spp. (E. siraeum, E. eligens and two unclassified Eubacterium MLGs) and Roseburia spp. (Tables S2 and S3). Similar findings were reported in previous studies on a variety of chronic diseases, including type 2 diabetes [3], liver cirrhosis [15], Crohn's disease [16] and ulcerative colitis [17]. These bacteria can produce lactate or butyrate, which could regulate gut permeability and induce the gut inflammatory response that precedes the development of diabetes [18, 19]."

* Page8, line27: In my opinion, it is better to introduce the reverse approach, I mean, that the GDM patients have these bacteria decreased. This manuscript is about the GDM patients, not about the healthy subjects.
# Reply: We thank the reviewer for this suggestion, and we have re-organized the discussion section and rewritten this sentence. Please refer to page 8, line3-9:
"The decreased microbes in GDM patients included Bifidobacterium spp. (including B. pseudocatenulatum, B. animalis and one unclassified MLG), Eubacterium spp. (E. siraeum, E. eligens and two unclassified Eubacterium MLGs) and Roseburia spp. (Tables S2 and S3). Similar findings were reported in previous studies on a variety of chronic diseases, including

type 2 diabetes [3], liver cirrhosis [15], Crohn's disease [16] and ulcerative colitis [17]. These bacteria can produce lactate or butyrate, which could regulate gut permeability and induce the gut inflammatory response that precedes the development of diabetes [18, 19]."

* Page8, line29: "… contribute to the pathogenesis of GDM" Why? you have assessed the functional analysis of these samples, you have the necessary data to establish a metabolic pathway for that.
# Reply: Please see the response to the next comments

* Page8, line54: "This result suggest that they work cooperatively…" How? the authors should propose a pathway.
# Reply: To address this question on how alterations in the microbiome contribute to the pathogenesis of GDM, we conducted a pathway analysis and proposed a potential mechanism that explain our results in Figure 7. In summary, we demonstrated an important association between the gut microbiota dysbiosis, functional changes and GDM.

* Page8, line56: In the result section, the authors established several relationships among particular bacteria and glucose. Please, explain and discuss it.
# Reply: We have added statements to clarify this point in the fourth paragraph of Discussion. Please refer to page 8, line 10-12; page 8,line 25- 30:
"Our data demonstrated the ratio of gross abundances of the GDM-enriched to control-enriched MLGs was positively correlated with blood glucose tolerance levels, suggesting that microbiome dysbiosis might have a direct association with GDM pathophysiology.
The functional analysis in the present study found that membrane transport, energy metabolic and PTS pathways were enriched in the GDM patients. PTS pathways are responsible for transporting glucose through outer and inner membranes and catalyzing the uptake of carbohydrates. The increased relative abundance of these pathways may indicate gut environment of a GDM status may stimulate bacterial accelerated usage of glucose as energy."

* Page9, line19: Antibiotic treatment is very important. If you have patients who took antibiotics, in the last three months, you must eliminate from the manuscript
# Reply: Thank you for your reminding. We have reviewed the data and confirmed that all participants did not take antibiotics during pregnancy (in the last ~5 months).

* Page10, line 7: Maybe one month is not enough, and more in a pregnancy situation, when the hormonal millie is constantly changing the environment
# Reply: We have reviewed the data and confirmed that all participants did not take antibiotics during pregnancy (in the last ~5 months).

* Page10, line8: prebiotics or probiotics?
# Reply: Our revision has addressed the confused description. Please refer to Page 9, line 21-26:
"The inclusion criteria of current study was as follows: 1) without diseases which might affect glucose metabolism or microbiome composition such as pre-pregnancy diabetes, hypertension, thyroid disorders, asthma, lipid metabolic disorders, inflammatory bowel disease, irritable bowel

syndrome and celiac disease; 2) had not received any antibiotic treatment 1 month before sample collection; 3) had not taken probiotics 2 weeks before sample collection.”

* Page10, line46: It would be necessary to include a table with the clinical information of the patients in order to know the metabolic health.
# Reply: Basic characteristics of the 124 pregnant women included in the study are summarized in new Table S1. In this study, we did not include women with pre-pregnancy diabetes, hypertensive disorders, thyroid disorders and asthma (please see the inclusion criteria in Page 9, line 21-26).

* Page10, line52: The recommendation is about 180-200 mg
# Reply: Thank you for identifying this inaccuracy. We have rechecked the instructions and our experimental records, confirmed that we used about 200 mg frozen feces. We have corrected this mistake in the manuscript. (Page 10, line 17)

* Page12, line 29: It is a usual assumption to use Shannon and Chao1 index to establish the alpha-diversity, due to both indexes study the richness and evenness in a different way.
# Reply: Thank you for your reminding. We used Shannon index and gene count (GC) to evaluate the alpha-diversity in our samples. In deep-sequenced metagemomic studies, the GC is generally used in deep-sequenced metagenomic samples [20].

Reference

1. Qiu X, Lu J-H, He J-R, Lam K-bH, Shen S-Y, Guo Y, Kuang Y-S, Yuan M-Y, Qiu L, Chen N-N: The Born in Guangzhou Cohort Study (BIGCS). 2017.
2. Arumugam M, Raes J, Pelletier E, Le Paslier D, Yamada T, Mende DR, Fernandes GR, Tap J, Bruls T, Batto JM et al: Enterotypes of the human gut microbiome. Nature 2011, 473(7346):174-180.
3. Qin J, Li Y, Cai Z, Li S, Zhu J, Zhang F, Liang S, Zhang W, Guan Y, Shen D et al: A metagenome-wide association study of gut microbiota in type 2 diabetes. Nature 2012, 490(7418):55-60.
4. Feng Q, Liang S, Jia H, Stadlmayr A, Tang L, Lan Z, Zhang D, Xia H, Xu X, Jie Z et al: Gut microbiome development along the colorectal adenoma-carcinoma sequence. Nature communications 2015, 6:6528.
5. Koren O, Goodrich JK, Cullender TC, Spor A, Laitinen K, Backhed HK, Gonzalez A, Werner JJ, Angenent LT, Knight R et al: Host remodeling of the gut microbiome and metabolic changes during pregnancy. Cell 2012, 150(3):470-480.
6. Sun L, Yu Z, Ye X, Zou S, Li H, Yu D, Wu H, Chen Y, Dore J, Clement K et al: A marker of endotoxemia is associated with obesity and related metabolic disorders in apparently healthy Chinese. Diabetes care 2010, 33(9):1925-1932.
7. Cani PD, Neyrinck AM, Fava F, Knauf C, Burcelin RG, Tuohy KM, Gibson GR, Delzenne NM: Selective increases of bifidobacteria in gut microflora improve high-fat-diet-induced diabetes in mice through a mechanism associated with endotoxaemia. Diabetologia 2007,

50(11):2374-2383.
8. Jayashree B, Bibin YS, Prabhu D, Shanthirani CS, Gokulakrishnan K, Lakshmi BS, Mohan V, Balasubramanyam M: Increased circulatory levels of lipopolysaccharide (LPS) and zonulin signify novel biomarkers of proinflammation in patients with type 2 diabetes. Molecular and cellular biochemistry 2014, 388(1-2):203-210.
9. Manco M, Putignani L, Bottazzo GF: Gut microbiota, lipopolysaccharides, and innate immunity in the pathogenesis of obesity and cardiovascular risk. Endocrine reviews 2010, 31(6):817-844.
10. Abreu MT: Toll-like receptor signalling in the intestinal epithelium: how bacterial recognition shapes intestinal function. Nature reviews Immunology 2010, 10(2):131-144.
11. Brun P, Castagliuolo I, Di Leo V, Buda A, Pinzani M, Palu G, Martines D: Increased intestinal permeability in obese mice: new evidence in the pathogenesis of nonalcoholic steatohepatitis. American journal of physiology Gastrointestinal and liver physiology 2007, 292(2):G518-525.
12. Garrett WS, Gallini CA, Yatsunenko T, Michaud M, DuBois A, Delaney ML, Punit S, Karlsson M, Bry L, Glickman JN et al: Enterobacteriaceae act in concert with the gut microbiota to induce spontaneous and maternally transmitted colitis. Cell host & microbe 2010, 8(3):292-300.
13. Liu J, Yan Q, Luo F, Shang D, Wu D, Zhang H, Shang X, Kang X, Abdo M, Liu B et al: Acute cholecystitis associated with infection of Enterobacteriaceae from gut microbiota. Clinical microbiology and infection : the official publication of the European Society of Clinical Microbiology and Infectious Diseases 2015, 21(9):851 e851-859.
14. Fei N, Zhao L: An opportunistic pathogen isolated from the gut of an obese human causes obesity in germfree mice. ISME J 2013, 7(4):880-884.
15. Qin N, Yang F, Li A, Prifti E, Chen Y, Shao L, Guo J, Le Chatelier E, Yao J, Wu L et al: Alterations of the human gut microbiome in liver cirrhosis. Nature 2014, 513(7516):59-64.
16. Gevers D, Kugathasan S, Denson LA, Vazquez-Baeza Y, Van Treuren W, Ren B, Schwager E, Knights D, Song SJ, Yassour M et al: The treatment-naive microbiome in new-onset Crohn's disease. Cell host & microbe 2014, 15(3):382-392.
17. Machiels K, Joossens M, Sabino J, De Preter V, Arijs I, Eeckhaut V, Ballet V, Claes K, Van Immerseel F, Verbeke K et al: A decrease of the butyrate-producing species Roseburia hominis and Faecalibacterium prausnitzii defines dysbiosis in patients with ulcerative colitis. Gut 2014, 63(8):1275-1283.
18. Peng L, Li ZR, Green RS, Holzman IR, Lin J: Butyrate enhances the intestinal barrier by facilitating tight junction assembly via activation of AMP-activated protein kinase in Caco-2 cell monolayers. The Journal of nutrition 2009, 139(9):1619-1625.
19. Vaarala O, Atkinson MA, Neu J: The "perfect storm" for type 1 diabetes: the complex interplay between intestinal microbiota, gut permeability, and mucosal immunity. Diabetes 2008, 57(10):2555-2562.
20. Le Chatelier E, Nielsen T, Qin J, Prifti E, Hildebrand F, Falony G, Almeida M, Arumugam M, Batto JM, Kennedy S et al: Richness of human gut microbiome correlates with metabolic markers. Nature 2013, 500(7464):541-546.
