# Supplementary material for: Connections between the human gut microbiome and gestational diabetes mellitus
Source: Gigascience. 2017 Jul 31;6(8):1–12. doi: 10.1093/gigascience/gix058 (PMC5597849; doi:10.1093/gigascience/gix058)
Supplement: Response-to-Reviewer-Comments_Revision-1.pdf [file gix058_Response-to-Reviewer-Comments_Revision-1.pdf]

Author's Response To Reviewer Comments

Reviewer reports:
Reviewer #1:
*Thanks to the authors for the thorough revision of the manuscript.
I have no further comments.
Reply: Thank you.

Reviewer #2:
The authors have addressed most of the changes and recommendations asked by the reviewers, and the manuscript has improved. However, there are still some concerns detailed below:

*The authors have included a table of the characteristics of the patients. However, they have not discussed any characteristics of the patients. For example, I have serious concerns about the age of the patients, which is significantly different between groups. GMD patients are older than healthy patients, and it must be discussed. Although it is a manuscript about gut microbiome, your conclusion should be taken into account the clinical parameters if you want to use the data for clinical purposes.
#Reply: Thank you for your comment. We have added the description of the basic characteristics of the study population in method section, please refer to page 10, line 13-15. We noted that the mean age between the two groups differed by 1.7 years. While statistically speaking, GDM patients are significantly older than healthy controls, we believe this small absolute difference is not clinically significant.

*Please, include in the limitations of the study the lack of serum samples from the patients, in order to measure LPS levels as this endotoxemia is the key aspect of your hypothesis.
#Reply: Thank you for your suggestion. We have added this limitation in our manuscript, please refer to page 9 line 23-25:
"Besides, due to the lack of serum samples, we could not measure LPS levels and describe the real endotoxemia level of the patients."

*Parabacteroides seems to have a key role in GDM, but the authors have not fully addressed this issue within the discussion.
#Reply: Thank you for your comment. From our results, Enterobacteriaceae, Parabacteroides, Megamonas and Phascolarctobacterium were found significantly enriched in GDM-patients, whereas Methanobrevibacter smithii, Alistipes spp., Bifidobacterium spp. and Eubacterium spp. were enriched in controls. These bacteria could play important roles together in GDM via facilitating LPS entry into the systemic circulation or inhibiting lactate or butyrate production. Several bacteria strains seem to have a key role; however, in our study, we suggested that microbiome dysbiosis (but not any particular bacterium) may have a direct association with GDM pathophysiology.

*The dysbiosis is usually assessed by a change in the richness of the groups. The authors have measured this index, but this information is not included in the results or discussion sections. However, they mentioned a dysbiosis in the first section of the results. Please, include this data.
#Reply: Thank you for your comment. We have added descriptions about the richness of the groups, please refer to page 4 line 29-30 to page 5 line 1-2:
"When we quantified the microbial (alpha) diversity within each subject, the GDM patients showed significantly lower gene count and Shannon index compared with the healthy pregnant women ($P < 0.05$ for both indexes, Mann-Whitney U test). "

*In the abstract, the "…" are not explicative. It should be fully understood by itself.
#Reply: With due respect we could not find “…” as you mentioned in our abstract. We would be happy to modify the sentence if you could point out where it is.
