# Supplementary material for: Connections between the human gut microbiome and gestational diabetes mellitus
Source: Gigascience. 2017 Jul 31;6(8):1–12. doi: 10.1093/gigascience/gix058 (PMC5597849; doi:10.1093/gigascience/gix058)
Supplement: Response-to-Reviewer-Comments_Revision-2.pdf [file gix058_Response-to-Reviewer-Comments_Revision-2.pdf]

Author's Response To Reviewer Comments

Dear Dr. Qiu,

Your manuscript "Connections between human gut microbiome and gestational diabetes mellitus" (GIGA-D-16-00167R2) has been assessed by our reviewers. Based on these reports, and my own assessment as Editor, I am pleased to inform you that it is potentially acceptable for publication in GigaScience, once you have carried out some essential revisions suggested by our reviewers.

Our curator, will also be in touch with you in order to get the supporting data ready for GigaDB.

The reviewers are happy with the scientific content; however, this paper still needs some final minor edits so that it conforms with our journal and article requirements for research. I have attached a new marked up version of the paper - please ensure you make the following edits:

#1) Potential Implications - move this section to after Discussion then renumber all the references in the text and reference list.
* Response ： We have done as request.

#2) i have checked the SRA to see if data for accession ERP020710 is available - it is not yet release. Please make this public and available - we cannot publish your paper until this is done.
*Response: Thank you.We have released the data on 22nd June.

#3) Please add the section on Consent to Publish - Human Data.
*Response:We have done as request.

Please ensure you make edits to the attached manuscript with our comments.

Please also take a moment to check our website at http://giga.edmgr.com/ for any additional comments that were saved as attachments.

Once you have made the necessary corrections, please submit a revised manuscript online at:

http://giga.edmgr.com/

If you have forgotten your username or password please use the "Send Login Details" link to get your login information. For security reasons, your password will be reset.

Please include a point-by-point within the 'Response to Reviewers' box in the submission system. Please ensure you describe additional experiments that were carried out and include a detailed rebuttal of any

criticisms or requested revisions that you disagreed with. Please also ensure that your revised manuscript conforms to the journal style, which can be found in the Instructions for Authors on the journal homepage.

Reviewer reports:

#Reviewer #2: The authors have assessed the changes to the manuscript and it is ready for publish.

*Response:Thank you.
