# Supplementary material for: Connections between the human gut microbiome and gestational diabetes mellitus
Source: Gigascience. 2017 Jul 31;6(8):1–12. doi: 10.1093/gigascience/gix058 (PMC5597849; doi:10.1093/gigascience/gix058)
Supplement: Reviewer-1-Report-(Original-Submission).pdf [file gix058_Reviewer-1-Report-(Original-Submission).pdf]

**Reviewer Report**

**Title:** Connections between human gut microbiome and gestational diabetes mellitus

**Version:** Original Submission **Date:** 1/19/2017

**Reviewer name:** Marloes Nitert Dekker

**Reviewer Comments to Author:**

GIGA-D-16-00167 Connections between human gut microbiome and GDM

This article describes the first metagenomic analysis of the gut microbiome in women with and without GDM. It is well-conducted, has generated fascinating data and will be of interest to people within the microbiome field and also in the diabetes field.

Major comments

* Methods-participant description:The description of the participant cohort is missing some important information: Please add to the table: birth weight of the infants, gestational age at delivery and mode of delivery. In addition, please provide more information on whether other disease states some of which may affect either glucose metabolism or microbiome composition such as thyroid disorders, asthma especially when treated with glucocorticoids, inflammatory bowel disease were excluded from the study as well.
* There was a wide range in the gestational age at which fecal samples were collected and also when OGTTs were performed. Furthermore, the fecal samples were not consistently collected at the same timepoint as the OGTT. It is possible that women who had an OGTT at 21 weeks gestation were normoglycemic at that time but crossed the threshold later and would have had GDM if they had an OGTT at 28 weeks. Has this been checked and what was the reason for the wide range in timing of OGTT testing? For women who had a large difference between OGTT and decal sample, for instance C008 and C112, this may be especially important. Also for C189, no OGTT was performed, how was ascertained that she was C and not GDM?
* Methods—taxonomical classification of genes. The cut-off for genus identity at 85% and is much lower than what is commonly used: 95-97% of genus and is lower than what is usually considered the threshold for family and even order. Please explain why this threshold was used.
* Methods—statistical analysis. Since in house Perl scripts were used for the rarefraction analysis, have these scripts been validated against other scripts to ensure that they are valid?
* In Figure 1A, it is not clear what the bacteria are that are denoted in blue. These are interesting bacteria which have been associated with different functions. Does the denotation in blue mean that these are not associated with either GDM or control? If so, why are they included?
* In figure 2, the representation of the co-occurrence of MLGs in the two groups is not optimal. It is understandable that the bacterial names are not displayed in the figure but since they are only available in the supplementary material, figure 2 becomes almost meaningless since except for a few bacteria, the

other connections are black boxes (or perhaps more appropriately red and green circles). I would like to suggest to the authors to perhaps include as part of figure 2 a list of the bacteria representing the clusters either in the legend or in the figure itself. That would make the figure more informative as a stand alone figure and would obviate the need to find the information in the supplementary material.
* In figure 3, were the GDM-enriched MLGs also correlated with the glucose measures of the control individuals and vice versa? This would be interesting data since it would give an insight into whether the relationship of the bacteria to the glucose levels is independent of the disease state or part of the physiological process.
* It is possible that there is a difference in gut microbiome composition in those women with GDM that were diagnosed based on mainly on their Fasting glucose levels vs only their 1 or 2 hour levels. Has this been checked?
* It was my impression from figure 1b that Aggregibacter was enriched in the control women, however in figure 6c it is shown as enriched in GDM. Please check this and of course also for the other bacteria mentioned.
* It could be argued too that the model to predict GDM should be compared with a prediction model that is based on easy to measure clinical parameters including prepregnancy BMI, family history of diabetes, a glucose measure (either glucose or HbA1c), gestational weight gain, rather than just comparing it to a microbiome explanatory model. The explanatory model is very effective (explaining >90% of variation) but for a fair comparison, especially if gut microbiome composition would be used for prediction, would be against commonly used clinical parameters.
* Furthermore, since the samples were taken at the time of OGTT, taking a blood sample and measuring blood glucose could be argued is easier and cheaper. Also since this is a cross-sectional microbiome analysis, it is not clear from these results whether or not the women developing GDM developed the gut dysbiosis in pregnancy or whether it was present pre-pregnancy. I think that therefore the first and concluding paragraph of the discussion should be reworded to include this. This does not distract from the value of the study since it points to bacteria which may be implicated in the pathogenic process of GDM.

**Methods**

Are the methods appropriate to the aims of the study, are they well described, and are necessary controls included? Yes

**Conclusions**

Are the conclusions adequately supported by the data shown? Yes

**Reporting Standards**

Does the manuscript adhere to the journal's guidelines on minimum standards of reporting? NoChoose an item.

**Statistics**

Are you able to assess all statistics in the manuscript, including the appropriateness of statistical tests used? Yes, and I have assessed the statistics in my report.

**Quality of Written English**

Please indicate the quality of language in the manuscript: Acceptable

**Declaration of Competing Interests**

Please complete a declaration of competing interests, considering the following questions:

- Have you in the past five years received reimbursements, fees, funding, or salary from an organisation that may in any way gain or lose financially from the publication of this manuscript, either now or in the future?
- Do you hold any stocks or shares in an organisation that may in any way gain or lose financially from the publication of this manuscript, either now or in the future?
- Do you hold or are you currently applying for any patents relating to the content of the manuscript?
- Have you received reimbursements, fees, funding, or salary from an organization that holds or has applied for patents relating to the content of the manuscript?
- Do you have any other financial competing interests?
- Do you have any non-financial competing interests in relation to this paper?

If you can answer no to all of the above, write 'I declare that I have no competing interests' below. If your reply is yes to any, please give details below.

I declare that I have no competing interests.

I agree to the open peer review policy of the journal. I understand that my name will be included on my report to the authors and, if the manuscript is accepted for publication, my named report including any attachments I upload will be posted on the website along with the authors' responses. I agree for my report to be made available under an Open Access Creative Commons CC-BY license (http://creativecommons.org/licenses/by/4.0/). I understand that any comments which I do not wish to be included in my named report can be included as confidential comments to the editors, which will not be published.

I agree to the open peer review policy of the journal

To further support our reviewers, we have joined with Publons, where you can gain additional credit to further highlight your hard work (see: https://publons.com/journal/530/gigascience). On publication of this paper, your review will be automatically added to Publons, you can then choose whether or not to claim your Publons credit. I understand this statement.

Yes
