# Supplementary material for: Connections between the human gut microbiome and gestational diabetes mellitus
Source: Gigascience. 2017 Jul 31;6(8):1–12. doi: 10.1093/gigascience/gix058 (PMC5597849; doi:10.1093/gigascience/gix058)
Supplement: Reviewer-2-Report-(Original-Submission).pdf [file gix058_Reviewer-2-Report-(Original-Submission).pdf]

Reviewer Report

Title: Connections between human gut microbiome and gestational diabetes mellitus

Version: Original Submission **Date:** 1/26/2017

Reviewer name: Isabel Moreno-Indias

Reviewer Comments to Author:

The authors have done a good work studying the differences between healthy and gestational diabetes mellitus pregnant women. It is an interesting approach due to there are scarce data showing the connections between gut microbiota and GDM. However, the authors have not been able to build a story with the good results they have.

Although the authors stated "In this study, we used whole-metagenome shotgun sequencing analyses of the gut microbiome during pregnancy to explore associations between GDM and the composition and abundance of microbial taxonomic units and functional genes. The objective was to obtain a comprehensive understanding of the gut microbiome's role in the etiopathogenesis of GDM", the opinion of this reviewer is that this aim has not been accomplished. The authors lack in relating all the data together, showing only that these groups of bacteria increase or decrease, resulting in a classical study similar to the 16S done until the date. Thus, although the authors, using elegant bioinformatics approaches, have shown new and relevant data for the elucidation of the gestational diabetes mellitus, the result is a manuscript without the relevancy to be published in Gigascience, at least in this current form.

Some suggestions and comments are listed below:

* A general feeling on the whole manuscript is that the authors seem that always leave work to be done in the future. There are many sentences like this "This intriguing observation warrants further studies". These sentences are in part responsable of my opinion of the manuscript, due to many of these comments are easy to measure, or the authors already have the information.

* When human samples are analyzed, it is important to show the characteristics of the study subjects. Humans are not homogeneous experimental animals. Many variables may obstruct the actual result.

* The authors showed in the results section that LPS and PTS systems were associated to the glucose tolerance levels, but in the discussion section, the authors do not explain anything about this relationship.

Particular comments:

* Page5, line 2: What about the family level?

* Page5, line 34: What are MLGs? you have not indicated it
* Page5, line 58: pro or pre-gestational body mass?
* Page6, line 44: It would be interesting to measure LPS amount if you have plasma samples. In this way, the authors could establish this assumption
* Page6, line 48: This sentence (the last one) is part of the discussion section.
* Page7, line23: This is part of the conclussions
* Page7,lines 44-54: This information has already mentioned in the results section
* Page8, line8: Which are the metabolic roles of these bacteria? the authors have the information
* Page8,line27: In my opinion, it is better to introduce the reverse approach, I mean, that the GDM patients have these bacteria decreased. This manuscript is about the GDM patients, not about the healthy subjects.
* Page8, line29: "... contribute to the pathogenesis of GDM" Why? you have assessed the functional analysis of these samples, you have the necessary data to establish a metabolic pathway for that.
* Page8, line54: "This result suggest that they work cooperatively..." How? the authors should propose a pathway.
* Page8, line56: In the result section, the authors established several relationships among particular bacteria and glucose. Please, explain and discuss it.
* Page9, line19: Antibiotic treatment is very important. If you have patients who took antibiotics, in the last three months, you must eliminate from the manuscript
* Page10, line 7: Maybe one month is not enough, and more in a pregnancy situation, when the hormonal millie is constantly changing the environment
* Page10, line8: prebiotics or probiotics?
* Page10, line46: It would be necessary to include a table with the clinical information of the patients in order to know the metabolic health.
* Page10, line52: The recommendation is about 180-200 mg
* Page12, line 29: It is a usual assumption to use Shannon and Chao1 index to establish the alpha-diversity, due to both indexes study the richness and evenness in a different way

-

Methods

Are the methods appropriate to the aims of the study, are they well described, and are necessary controls included? Yes

Conclusions

Are the conclusions adequately supported by the data shown? Yes

Reporting Standards

Does the manuscript adhere to the journal's guidelines on [minimum standards of reporting?]() YesChoose an item.

Statistics

Are you able to assess all statistics in the manuscript, including the appropriateness of statistical tests used? No, and I do not feel adequately qualified to assess the statistics.

Quality of Written English

Please indicate the quality of language in the manuscript: Acceptable

Declaration of Competing Interests

Please complete a declaration of competing interests, considering the following questions:

- Have you in the past five years received reimbursements, fees, funding, or salary from an organisation that may in any way gain or lose financially from the publication of this manuscript, either now or in the future?
- Do you hold any stocks or shares in an organisation that may in any way gain or lose financially from the publication of this manuscript, either now or in the future?
- Do you hold or are you currently applying for any patents relating to the content of the manuscript?
- Have you received reimbursements, fees, funding, or salary from an organization that holds or has applied for patents relating to the content of the manuscript?
- Do you have any other financial competing interests?
- Do you have any non-financial competing interests in relation to this paper?

If you can answer no to all of the above, write 'I declare that I have no competing interests' below. If your reply is yes to any, please give details below.

I declare that I have no competing interests

I agree to the open peer review policy of the journal. I understand that my name will be included on my report to the authors and, if the manuscript is accepted for publication, my named report including any attachments I upload will be posted on the website along with the authors' responses. I agree for my report to be made available under an Open Access Creative Commons CC-BY license (http://creativecommons.org/licenses/by/4.0/). I understand that any comments which I do not wish to be included in my named report can be included as confidential comments to the editors, which will not be published.

I agree to the open peer review policy of the journal

To further support our reviewers, we have joined with Publons, where you can gain additional credit to further highlight your hard work (see: https://publons.com/journal/530/gigascience). On publication of

this paper, your review will be automatically added to Publons, you can then choose whether or not to claim your Publons credit. I understand this statement.

Yes
